# *HvarAKR1B1* ‐mediated tolerance in *Hippodamia variegata* : Deciphering the metabolic adaptation and fitness costs under prolonged starvation

**DOI:** 10.1002/ps.70851

**Published:** 2026-04-23

**Authors:** Bing‐mei Song, De‐qin Hu, Yuan Li, Wang‐quan Jiao, Xiao‐li Ma, Hong‐sheng Pan

**Affiliations:** ^1^ Plant Protection Scientific Observation and Research Station in Bayingol of the Ministry of Agriculture and Rural Affairs Institute of Plant Protection, Xinjiang Uygur Autonomous Region Academy of Agricultural Sciences Urumqi China; ^2^ College of Life Science and Technology Xinjiang University/Xinjiang Key Laboratory of Biological Resources and Genetic Engineering Urumqi China

**Keywords:** *Hippodamia variegata*, starvation stress, integrated transcriptome and metabolome analysis, *HvarAKR1B1*, RNA interference

## Abstract

**BACKGROUND:**

*Hippodamia variegata* is an important natural enemy of pests in cotton fields in Xinjiang, China. In natural settings, this species frequently encounters food scarcity due to environmental fluctuations, seasonal shifts, and the uneven distribution of prey. However, studies on the physiological and molecular responses of *Hippodamia variegata* to starvation stress remain scarce.

**RESULTS:**

This study found that starvation stress shortened developmental duration, reduced survival rate, and decreased fecundity of *Hippodamia variegata*. Transcriptomic and metabolomic analyses identified 337 differentially expressed genes (DEGs) and 764 differentially abundant metabolites (DAMs) in response to starvation. Integrated omics analysis indicated the significant enrichment of energy metabolism pathways related to carbohydrates, amino acids, and lipids. Weighted gene co‐expression network analysis (WGCNA) based on RNA‐sequencing data suggested the potential involvement of gene721 (*HavrAKR1B1*) in the starvation response. The full‐length open reading frame (ORF) of *HavrAKR1B1* spanned 948 bp and encoded a protein of 315 amino acids. The expression level of *HavrAKR1B1* remained low from the egg to the third instar larvae, increased from the fourth instar to the pupal stage. RNA interference (RNAi) showed that silencing *HvarAKR1B1* significantly shortened developmental duration and survival rate, and led to a substantial decline in starvation tolerance.

**CONCLUSION:**

These findings indicate that although starvation stress adversely affects the development of *Hippodamia variegata*, it can prompt a self‐protection response by up‐regulating glucose metabolism‐related gene *HavrAKR1B1*. This study enhanced our understanding of the ecological adaptability of *Hippodamia variegata* and offered a novel theoretical basis for improving biological control strategies. © 2026 The Author(s). *Pest Management Science* published by John Wiley & Sons Ltd on behalf of Society of Chemical Industry.

## Graphical abstract

Starvation stress significantly affected the growth and development of *Hippodamia* *variegata*. Combined transcriptome and metabolome analysis and RNA interference verification revealed that *HvarAKR1B1* may be involved in the response of *Hippodamia variegata* to starvation stress.

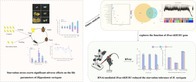

## 1 INTRODUCTION

*Aphis gossypii* Glover (Hemiptera: Aphididae) is a globally distributed agricultural pest that inflicts severe economic losses on global crop production through direct sap‐feeding, transmission of plant viruses, and excretion of honeydew.^1^, ^2^ Chemical control has long been the primary strategy for managing *A. gossypii*.^3^, ^4^ However, excessive reliance on pesticides has not only led to the development of resistance against various pesticides, but also caused environmental contamination, posing a threat to human health and non‐target organisms.^5^, ^6^ In light of these challenges, the employment of natural enemies has emerged as a critical component in the environmentally sustainable management of *A. gossypii*.^7^

*Hippodamia variegata* (Coccinellidae: Coleoptera) is a prominent natural enemy of *A. gossypii* in cotton ecosystems because of its early seasonal occurrence, high predation capacity, and broad prey range.^8^, ^9^ However, factors such as environmental fluctuations,^10^, ^11^ seasonal shifts,^12^ and uneven prey availability^13^ often subject insects to intermittent nutritional stress, particularly during metamorphosis.^14^ Studies indicate that starvation can significantly impact the physiology and behavior of ladybirds, thereby influencing their development, reproduction, and survival, which may ultimately compromise their efficacy as biological control agents.^15^, ^16^ Despite its ecological importance, the physiological and molecular mechanisms underlying the response of *Hippodamia variegata* to starvation stress remain poorly understood. Therefore, further elucidation of its adaptation mechanism under starvation stress not only facilitates comprehension of its ecological adaptability and stress resistance strategies but also provides a theoretical foundation for optimizing field conservation and enhancing biological control efficacy.^17^

To cope with starvation stress, insects activate a series of physiological and biochemical adaptations to conserve energy and maintain homeostatic balance, including reduced activity levels, suppressed metabolic rates, and preservation of structural and functional integrity in key tissues.^16^, ^18^, ^19^ Studies have demonstrated that prolonged periods of nutrient deprivation result in a significant decline in energy reserves across a range of insect species.^20^, ^21^, ^22^ For instance, in *Harmonia axyridis*, trehalose levels progressively decrease with extended periods of starvation.^23^ Similarly, *Moina macrocopa* displays substantial decreases in glucose, total soluble protein, and total amino acid content following 24 and 48 h of starvation.^24^ In *Drosophila virilis*, 24 h starvation impairs juvenile hormone degradation, delays oocyte maturation, induces the degradation of early vitellogenic egg chambers, leads to the accumulation of mature oocytes, and results in the cessation of oviposition.^25^ Furthermore, in *Schistocerca americana*, starvation significantly suppresses juvenile hormone synthesis, thereby compromising reproductive capacity, an effect that remains only partially reversible even after refeeding.^26^

In addition to adaptive physiological and reproductive changes, insects initiate complex molecular responses to starvation. For example, in *Panonychus citri*, starvation significantly up‐regulates the expression of fatty acid binding protein, a key gene involved in lipid mobilization, thereby extending survival duration.^27^ In *Cyrtorhinus lividipennis*, starvation tolerance is enhanced by the suppression of reproduction‐related genes such as S6K, as well as components of the insulin and TOR signaling pathway.^28^ In *Bombyx mori*, starvation up‐regulates β‐oxidation‐related genes while inhibiting fatty acid synthesis, alongside enhanced antioxidant capacity and activation of the pentose phosphate pathway.^29^ Furthermore, in *Spodoptera litura*, starvation leads to significant down‐regulation of glycogen synthase and glycogen phosphorylase levels, as well as reduced expression of glycolysis and tricarboxylic acid cycle‐related genes.^30^

In this study, we investigated the developmental duration, fecundity, and survival rate of *Hippodamia variegata* under starvation stress, providing a systematic assessment of the fitness costs associated with nutritional deprivation in this species. Using transcriptomic and metabolomic techniques, differentially expressed genes (DEGs) and differentially abundant metabolites (DAMs) in *Hippodamia variegata* were identified under both starved and normally fed conditions. Weighted gene co‐expression network analysis (WGCNA) identified *AKR1B1* as a key gene involved in the response to starvation stress. The *HvarAKR1B1* gene from *Hippodamia variegata* (*HvarAKR1B1*) was successfully cloned, and its physicochemical properties were characterized. The spatiotemporal expression patterns of *HvarAKR1B1* were analyzed using quantitative real‐time polymerase chain reaction (RT‐qPCR). Furthermore, the function of *HvarAKR1B1* in response to starvation stress was evaluated using RNA interference (RNAi) technology. These findings provide a new perspective for understanding the function of *HvarAKR1B1* in the adaptation of natural enemy insects to starvation stress and lay the foundation for the optimization of field conservation strategies and improvement of biological control efficacy.

## 2 MATERIALS AND METHODS

### 2.1 Insects rearing

*Hippodamia variegata* was stably reared in our laboratory using *A. gossypii* at the Plant Protection Scientific Observation and Research Station in Bayingol of the Ministry of Agriculture and Rural Affairs (Korla, Bayingol Mongolian Autonomous Prefecture, Xinjiang Uygur Autonomous Region, China; 41.75° N, 85.81° E). Larvae were cultured at 25 °C, 65% relative humidity, and a photoperiod of 14 h:10 h light/dark. No insecticide exposure was observed during the rearing period.

### 2.2 Effects of starvation stress on the fitness of *Hippodamia variegata*

Fifteen single‐pair matings of *Hippodamia variegata* (F_0_ generation) were established. Egg masses were collected daily during the peak oviposition period (5–8 days after pairing) and incubated under uniform conditions. Two experimental treatments were applied: starvation stress and normal feeding (control group). For the control group the aphid supply was set at a level close to the reported maximum daily consumption for each developmental stage (approximately 30, 50, 130, 180, and 250 aphids for first, second, third, fourth instar larvae, and adults, respectively).^31^ In the starvation group, the aphid supply was maintained at 25% of that in the control group (for the first, second, third, fourth instar larvae and adults, approximately 8, 13, 30, 45, and 62 aphids, respectively). The 25% supply level was calculated as a severe but sustainable deficit relative to the established maximum consumption capacity, effectively modeling prolonged prey scarcity. Each treatment included three replicates, with 140 newly hatched larvae per replicate. The larvae were individually reared in numbered glass beakers. Survival rates, larval instar development, pupation, and adult emergence were systematically recorded daily at 10:00 a.m. After sex identification of F_1_ adults using a stereomicroscope (S9i; Leica, Heerbrugg, Switzerland), single‐pair matings were conducted, with each pair housed in a separate glass beaker and provided with aphids, as described earlier. Following the onset of oviposition, the number of eggs laid per female during the first 10 days was recorded, and F_2_ eggs were collected daily.

### 2.3 Transcriptomic analysis of *Hippodamia variegata*

For transcriptomic analysis, samples were collected separately from the starvation‐stressed and control groups (the treatment protocols for both are described in Section 2.2). Three independent biological replicates were prepared for each treatment group. To obtain a sufficient amount of RNA, each replicate comprised a pooled sample containing individuals from all developmental stages of *Hippodamia variegata*. Specifically, one pooled biological replicate consisted of 100 eggs, 25 first instar larvae, 20 second instar larvae, 15 third instar larvae, ten fourth instar larvae, three pupae, three female adults, and three male adults from the same treatment group. All samples were rapidly frozen in liquid nitrogen and stored at −80 °C. Total RNA was extracted using TRIzol reagent (TransGen Biotech, Beijing, China) according to the manufacturer's instructions. The RNA samples were subsequently sent to Beijing Biomarker Biotechnology Co., Ltd (Beijing, China) for library construction and sequencing (Illumina NovaSeq 6000 platform to generate 150 bp paired‐end reads; Illumina, San Diego, CA, USA). Complementary DNA (cDNA) libraries were constructed using the NEBNext® UltraTM RNA Library Prep, and the size distribution of the library (300–500 bp) was verified using an Agilent 2100 Bioanalyzer (Agilent Technologies, Santa Clara, CA, USA). Subsequently, the quantified libraries were sequenced on an Illumina platform (PE150 mode) to generate at least 40 million high‐quality reads for each sample. Total RNA extraction, library preparation, and transcriptome sequencing were commissioned by Beijing Biomarker Technology Co., Ltd.

Differential expression analysis between groups was performed using BMKCloud (Beijing Biomarker Technology Co., Ltd). The quality of the raw sequence reads was assessed using the FastQC tool.^32^ Adapter sequences and low‐quality bases were trimmed using Trimmomatic software. The process of genome alignment was conducted utilizing the HISAT2 software (version 2.0.4).^33^ StringTie software was used for transcript assembly and quantification of gene expression levels. StringTie‐generated GTF files containing transcript information were processed to create gene‐level FPKM (fragments per kilobase million) tables. The DEGs were then subjected to screening using edgeR (version 3.32.1) software,^34^ with the criteria set at Fold Change ≥ 1.5 and *P* < 0.05. Finally, enrichment analysis was performed using R/cluster Profiler (version 4.4.4) and R/topGO (version 2.48.0).^35^

### 2.4 Metabolomic analysis of *Hippodamia variegata*

For metabolomic analysis, the sampling method was similar to that described in Section 2.2. Three independent biological replicates were prepared for both the starvation‐stressed and control groups. To ensure sufficient material for analysis and to obtain a representative metabolic profile across development, each replicate consisted of a pooled sample covering all developmental stages of *Hippodamia variegata*. Specifically, one biological replicate included 100 eggs, 25 first instar larvae, 20 second instar larvae, 15 third instar larvae, ten fourth instar larvae, three pupae, three female adults, and three male adults, all collected from the same treatment group. All individuals within a replicate were ground together into a homogeneous powder. Subsequently, a 30 mg aliquot was taken from each powdered replicate for metabolomic sequencing, while the remaining sample was stored for potential future use. The separation and quantification of metabolites were conducted by Beijing Biomarker Biotechnology Co., Ltd (Beijing, China) using liquid chromatography–mass spectrometry (LC–MS). Each sample was mixed with 1000 μL of extraction solution consisting of methanol, acetonitrile, and water in a ratio of 1:2:1 (*v/v/v*), followed by vortex mixing for 30 s (Model TL2020; Bionoon, Shanghai, China). Subsequently, the samples were homogenized using a grinding instrument at 45 Hz for 10 min (Model TL‐3000; Bionoon, Shanghai, China). The samples were then subjected to ultrasonication in an ice‐water bath for 10 min and incubated at −20 °C for 1 h. After incubation, the samples were centrifuged at 11 000 × *g* for 15 min at 4 °C (Model GL0650R; Monad, Wuhan, China). A 300 μL aliquot of the supernatant was filtered through a 0.22 μm organic‐phase nylon membrane into a 2 mL autosampler vial for analysis. For quality control (QC), 10 μL of each sample was pooled to prepare a QC sample for online analysis. Chromatographic separation was performed on a Waters ACQUITY UPLC BEH C18 column (1.7 μm, 2.1 mm × 100 mm; Waters, Milford, MA, USA), and the column temperature was maintained at 45 °C. The mobile phase consisted of 0.1% formic acid and 5 mm ammonium acetate (A) and 0.1% formic acid (B); and in the negative mode, the mobile phase consisted of 0.1% formic acid and 5 mm ammonium acetate (A), 0.1% formic acid (B). The raw data files generated by ultrahigh‐performance liquid chromatography–tandem mass spectrometry (UHPLC–MS/MS) were processed using Compound Discoverer 3.3 for peak alignment, peak picking, and quantification of each metabolite. Principal component analysis (PCA), partial least squares‐discriminant analysis (PLS‐DA), and orthogonal partial least squares‐discriminant analysis (OPLS‐DA) were performed using the Biomarker online platform. Subsequently, a multivariate analysis model was constructed based on OPLS‐DA to preliminarily screen for DAMs between the starvation‐stressed and control groups, with the variable importance in projection (VIP) threshold set to 1, the cross‐validation folds set to 3, and the number of permutation tests set to 200. For clustering heat maps, the data were plotted using the heatmap package in R 4.3.1. The functions of these metabolites and metabolic pathways were studied using the Kyoto Encyclopedia of Genes and Genomes (KEGG) database. The metabolic pathway enrichment of DAMs was performed, and when the *P*‐value of the metabolic pathway was < 0.05, metabolic pathways were considered statistically significantly enriched.

### 2.5 Integrated transcriptomic and metabolomic analysis of *Hippodamia variegata*

The transcriptome and metabolome were comprehensively analyzed using BMKCloud. Briefly, the DEGs and DAMs within the same pathway were simultaneously mapped onto the KEGG pathway database. Subsequently, the expression patterns of the genes and metabolites in each KEGG pathway were analyzed.

Co‐expression networks were constructed using the WGCNA package in R (version 1.47) to obtain highly correlated genes involved in the response to starvation stress.^36^ Transcriptome data were filtered using 11 938 genes to construct co‐expression modules. The power was 13, TOMType was unsigned, mergeCutHeight was 0.1, and minModuleSize was 50.

### 2.6 RNA extraction, cDNA synthesis, and RT‐qPCR analysis

Total RNA was extracted from different developmental stages and tissues of female and male adults of *Hippodamia variegata* using the TransZol Up Plus RNA Kit (TransGen Biotech) according to the manufacturer's protocol, and 1 μg total RNA was used to synthesize the first‐strand cDNA using the TransScript One‐Step gDNA Removal (TransGen Biotech). RT‐qPCR was performed using an ABI Prism 7500 system (Applied Biosystems, Foster City, CA, USA) to assess the relative expression of the target genes. Each 20 μL reaction mixture comprised 10 μL of 2 × PerfectStart® Green qPCR SuperMix (Tiangen Biotech, Beijing, China), 0.4 μL of each gene‐specific primer (10 μm), 1 μL of cDNA template, 7.8 μL of nuclease‐free water, and 0.4 μL of 50 × ROX dye. The thermal cycling protocols were as follows: denaturation at 95 °C for 10 min, followed by 40 cycles of denaturation at 95 °C for 10 s, annealing at 58 °C for 30 s, and extension at 72 °C for 30 s. Gene‐specific primers for RT‐qPCR were designed using Primer Premier 5.0 software, and the primer sequences are listed in Supporting Information Table S1. *Elongation factor 1‐alpha* (*EF1α*) was selected as the internal reference gene for the normalization. The relative expression levels were calculated using the 2^−△△Ct^ method.^37^, ^38^

### 2.7 *HvarAKR1B1* gene cloning and sequence analysis

The coding sequence (CDS) of *HvarAKR1B1* was identified from our transcriptome database and amplified using gene‐specific primers (Table S1). The resulting PCR product was ligated into the pEasy‐T1 vector and introduced into Trans‐T1 competent cells (TransGen Biotech). Positive clones were randomly selected and sequenced by Sangon Biotech (Shanghai, China). The physicochemical properties of the deduced proteins were predicted using the ExPASy Proteomics Server (https://web.expasy.org/compute_pi/). For phylogenetic analysis, a neighbor‐joining (1000 bootstrap replicates) tree was constructed using MEGA 11.0 software.

### 2.8 Spatio‐temporal expression profile of *HvarAKR1B1* in *Hippodamia variegata*

Samples from different developmental stages were collected as described in Section 2.3. Additionally, tissues, including the head, thorax, abdomen, feet, and wings, were dissected from both male and female adults. For each tissue type, three biological replicates were established, with each replicate consisting of a pooled sample derived from the same tissue of 15 same‐sex adults. Total RNA extraction and cDNA synthesis for all samples were performed as described in Section 2.6. The expression levels of *HvarAKR1B1* in *Hippodamia variegata* were analyzed using RT‐qPCR.

### 2.9 Double‐stranded RNA synthesis and RNAi assays

To validate the function of *HvarAKR1B1*, fragments of double‐stranded RNA (dsRNA) were designed from the open reading frame (ORF) of the *HvarAKR1B1* gene, with double‐stranded green fluorescent protein (ds*GFP*) used as a non‐target control in our RNAi experiments. The dsRNA was synthesized using the T7 RiboMAX Express RNAi System (Promega, Madison, WI, USA), according to the manufacturer's instructions. The specific primers used for dsRNA synthesis are listed in Table S1.

On the first day of the experiment, fourth instar larvae of *Hippodamia variegata* (larvae were reared under normal conditions and were not subjected to prior starvation stress) were microinjected with 1 μL of synthetic ds*HvarAKR1B1* (1000 ng/μL), while the control groups were injected with equal amounts of ds*GFP* using a Nanoject III Programmable Nanoliter Injector (Drummond Scientific, Broomall, PA, USA). The experimental design included three groups: non‐injection, ds*GFP*, and ds*HvarAKR1B1*, each consisting of three biological replicates with 35 larvae per replicate. To determine the silencing efficiency of ds*HvarAKR1B1*, samples were collected at 24, 48, and 72 h after injection, rapidly frozen in liquid nitrogen, and stored at −80 °C. Total RNA extraction and first‐strand cDNA synthesis were performed as described earlier. The transcription levels of *HvarAKR1B1* were analyzed using RT‐qPCR, as described earlier. This analysis included three biological replicates (ten larvae per replicate) and two technical replicates. *EF1α* was used as the reference gene for data normalization, and the relative expression levels were calculated using the 2^−△△Ct^ method.^37^

To assess the baseline fitness effects of *HvarAKR1B1* silencing, larvae were reared under standard non‐stressed conditions after dsRNA microinjection. Survival rates and development were observed and recorded every 12 h. Each treatment was repeated thrice, with each replicate containing 30 *Hippodamia variegata* individuals.

### 2.10 Statistical analysis

All statistical analyses were performed using SPSS 20.0 and R version 4.2.2. Before conducting parametric tests, the normality of all data (including life table parameters) was assessed using the Shapiro–Wilk test (or skewness‐kurtosis test). Homogeneity of variances was examined using Levene's test. For two‐group comparisons, if the data met both assumptions of normality and homogeneity of variance, an independent samples Student's *t*‐test was applied. If the data met normality but violated the assumption of homogeneity of variance, Welch's *t*‐test was used. For data that did not meet the assumption of normality, the non‐parametric Mann–Whitney *U* test was employed. The fitness cost was statistically analyzed using an independent sample Student's *t*‐test, while the Mann–Whitney *U* test was used for pre‐oviposition data (*: *P* < 0.05; **: *P* < 0.01; ***: *P* < 0.001; ****: *P* < 0.0001). The expression patterns of *HvarAKR1B1*, silencing efficiency, and developmental data were analyzed using one‐way analysis of variance (ANOVA), followed by Tukey's multiple range test.

## 3 RESULTS

### 3.1 Effects of starvation stress on the fitness of *Hippodamia variegata*

**Figure 1:**
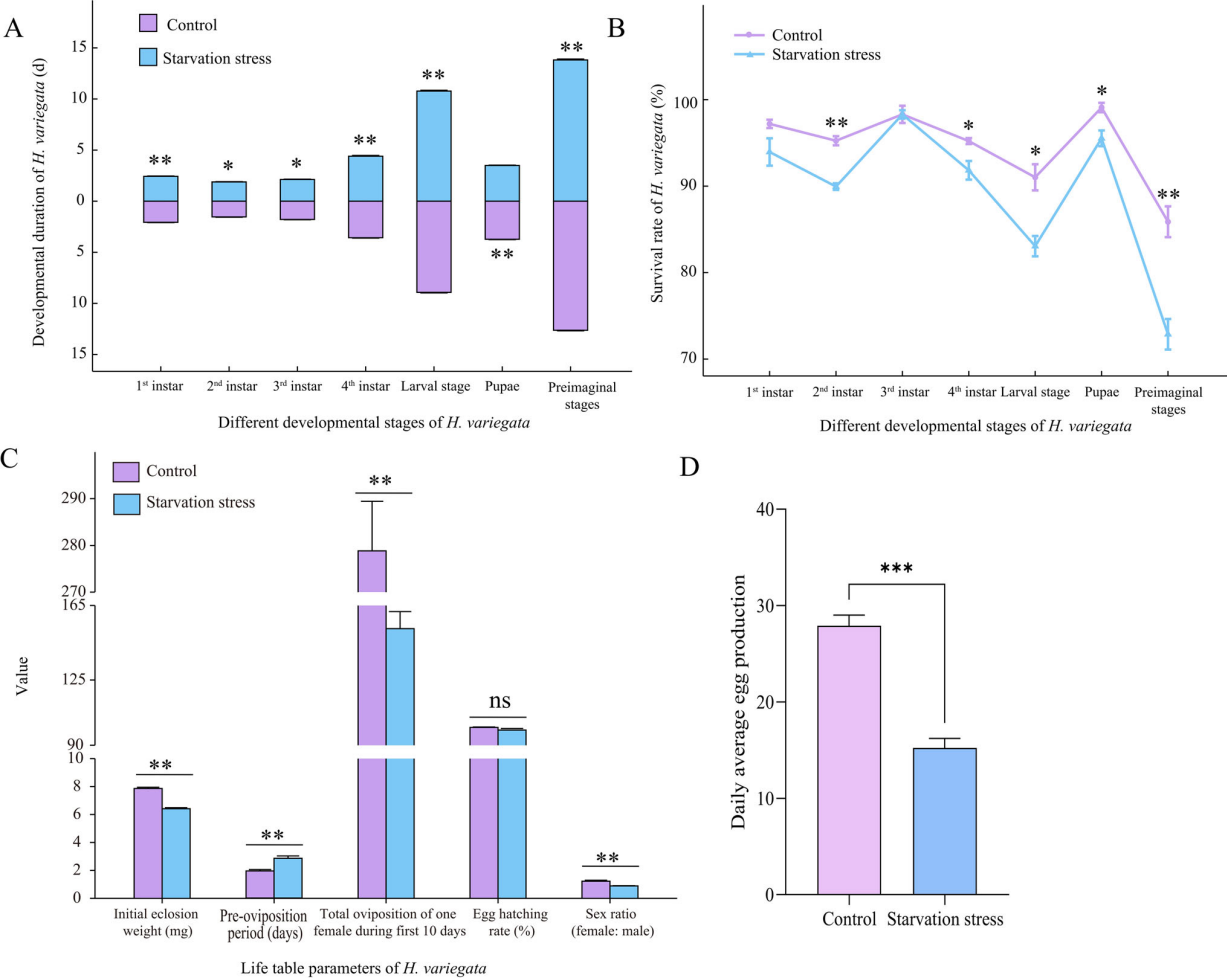
Effects of starvation stress on the fitness of *Hippodamia variegata*. (A) The effects of starvation stress on the developmental duration. (B) The effects of starvation stress on the survival rate. (C) The effects of starvation stress on certain life parameters. (D) The daily average egg production. The significant markers in the figure were analyzed using the Student's *t*‐test, and the pre‐oviposition data were analyzed using the Mann–Whitney *U* test. The symbols ‘*’ and ‘**’ indicate that the corresponding indices of *Hippodamia variegata* had significant differences (*P* < 0.05) and extremely significant differences (*P* < 0.01) between the starvation stress and control group, respectively. The notation ‘ns’ indicates that there is no significant difference between the two treatments (*P* > 0.05).

Starvation stress significantly affected the fitness of *Hippodamia variegata* (*P* < 0.05). Compared with the control group, starvation prolonged the developmental durations of the first to fourth instar larvae by 0.37 days (*t* = 12.80, df = 4, *P* < 0.001), 0.35 days (*t* = 8.45, df = 4, *P* = 0.01), 0.33 days (*t* = 4.32, df = 4, *P* = 0.012), and 0.76 days (*t* = 11.967, df = 4, *P* < 0.001), respectively. Consequently, the total larval and preimaginal stages increased by 1.81 days (*t* = 12.80, df = 4, *P* < 0.001) and 0.88 days (*t* = 12.80, df = 4, *P* < 0.001), respectively. Conversely, the pupal stage was significantly shortened by 0.40 days (*t* = 12.80, df = 4, *P* < 0.001) (Fig. 1(A)). Survival rates of first instar, second instar, and fourth instar larvae, as well as the overall larval and adult stages, decreased by 3.46% (*t* = 3.432, df = 4, *P* = 0.026), 4.43% (*t* = 4.836, df = 4, *P* = 0.008), 4.20% (*t* = 3.901, df = 4, *P* = 0.018), 7.31% (*t* = 4.548, df = 4, *P* = 0.010), and 12.38% (*t* = 5.136, df = 4, *P* = 0.007), respectively (Fig. 1(B)). Furthermore, reproductive performance was adversely affected. Starvation prolonged the female pre‐oviposition period by 0.91 days (*Z* = 4.64, *P* < 0.001). Total fecundity per female during the first 10 days (*t* = 9.04, df = 4, *P* < 0.001), daily average egg production (*t* = 14.72, df = 4, *P* < 0.001), emergence weight (*t* = 12.43, df = 4, *P* < 0.001), and sex ratio (*t* = 4.74, df = 4, *P* = 0.009) were all significantly reduced in the treatment group. However, the egg hatching rate did not differ significantly between the two groups (*t* = 0.789, df = 4, *P* = 0.474) (Fig. 1(C),(D)).

### 3.2 Starvation stress retards ovarian development

**Figure 2:**
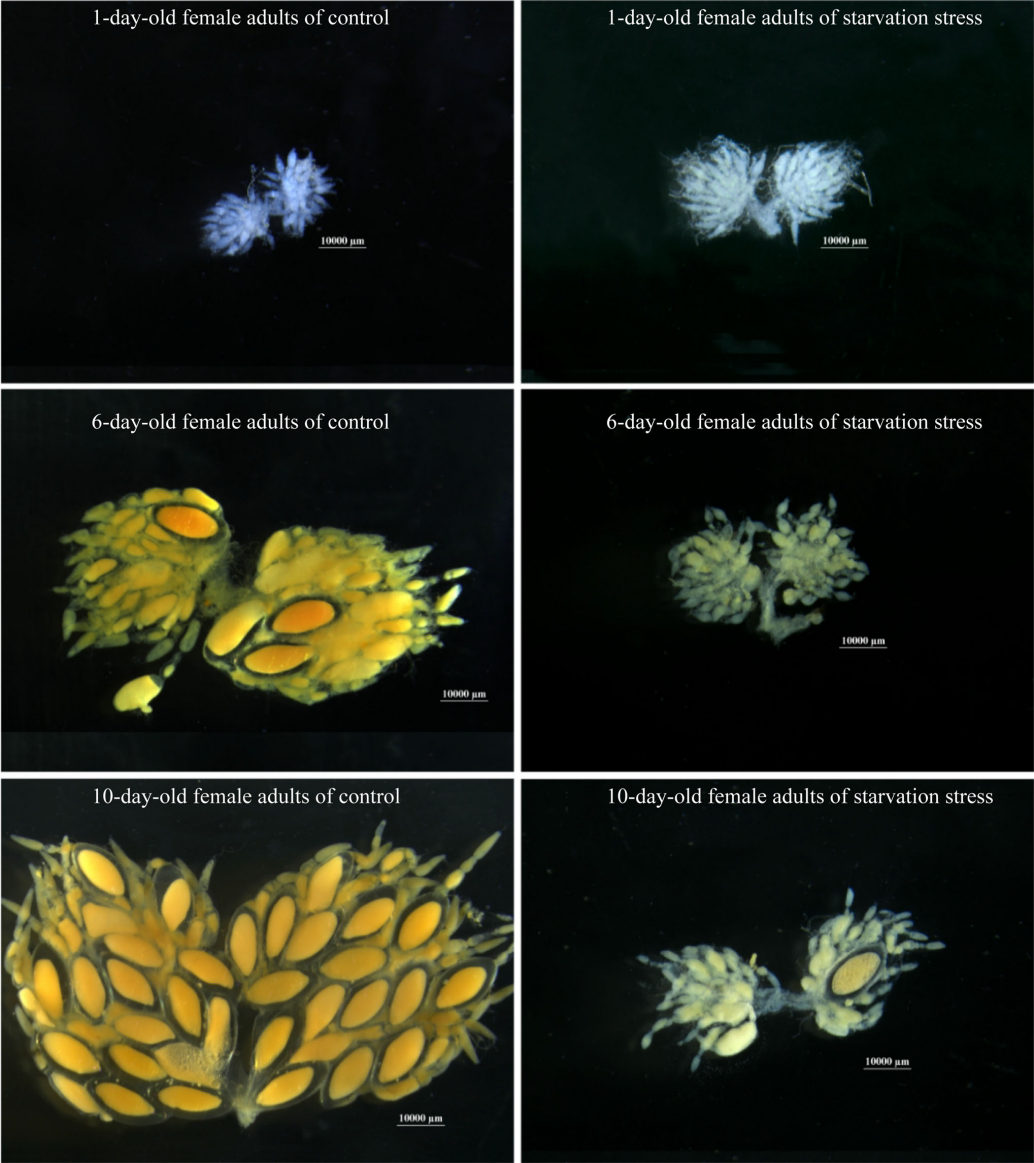
The effects of starvation stress on the ovarian development of *Hippodamia variegata*.

Ovarian development examination revealed that starvation stress led to the retardation of maturation. Under both control and starvation conditions, the egg chambers of *Hippodamia variegata* females formed within 1 day after eclosion, with no yolk deposition observed at this initial stage. By day 6, the egg chambers in the control group appeared bright yellow, exhibiting substantial yolk deposition and a limited number of mature eggs. In contrast, those in the starvation group appeared pale yellow, showed minimal yolk deposition, and did not contain mature eggs. On day 10, the control egg chambers remained bright yellow, characterized by abundant yolk deposition and a large number of mature eggs. Conversely, egg chambers in the starvation group were still pale yellow, indicating only a moderate increase in yolk deposition and the presence of a few mature eggs (Fig. 2).

### 3.3 Effect of starvation stress on the transcriptome of *Hippodamia variegata*

Transcriptomic analysis identified a total of 337 DEGs in *Hippodamia variegata* between the starvation stress and control groups, among which 124 were up‐regulated, and 213 were down‐regulated (Supporting Information Fig. S1(A)). To elucidate the biological functions of these DEGs, Gene Ontology (GO) enrichment and KEGG pathway analyses were performed. KEGG analysis indicated that the DEGs were significantly enriched in 50 metabolic pathways. The top 20 enriched pathways included linoleic acid metabolism (ko00591), arachidonic acid metabolism (ko00590), phagosome (ko04145), lysosome (ko04142), autophagy (ko04140), and Wnt signaling pathway (ko04310), among others (Fig. S1(B)). GO analysis revealed that 190 DEGs were enriched in 40 functional terms across the three major categories: 22 in biological processes, 13 in molecular functions, and five in cellular components (Fig. S2).

### 3.4 Effect of starvation stress on the metabolism of *Hippodamia variegata*

A total of 1270 metabolites were detected in *Hippodamia variegata*, including 354 lipids, 286 amino acids, 150 carbohydrates, 143 organic acids, 97 nucleotides, 76 alcohols, 73 ketoaldehyde esters, 22 vitamins, coenzymes, ten hormones, nine amines, seven cholines, seven bile acids, and 36 other metabolites. Comparative analysis between the starvation stress and control groups identified 764 DAMs, with 484 up‐regulated and 280 down‐regulated under starvation conditions (Fig. S3(A)). Among these, 40 DAMs were associated with carbohydrate metabolic pathways, 29 with amino acid metabolism, 15 with membrane transport, and 23 with nucleotide metabolism (Fig. S3(B)). Key enriched metabolic pathways included steroid hormone biosynthesis (map00140), sphingolipid metabolism (map00600), oxidative phosphorylation (map00190), sphingolipid signaling pathway (map04071), and fatty acid degradation (Fig. S3(C)). Notable differential metabolites included cholesterol‐D7, 8‐dehydrocholesterol, 4‐beta‐hydroxycholesterol, 20,22‐dihydroxycholesterol, estradiol hexanoate, progesterone NA progesterone, dehydroepiandrosterone, and estrone.

### 3.5 Integrated transcriptomic and metabolomic analysis of *Hippodamia variegata*

**Figure 3:**
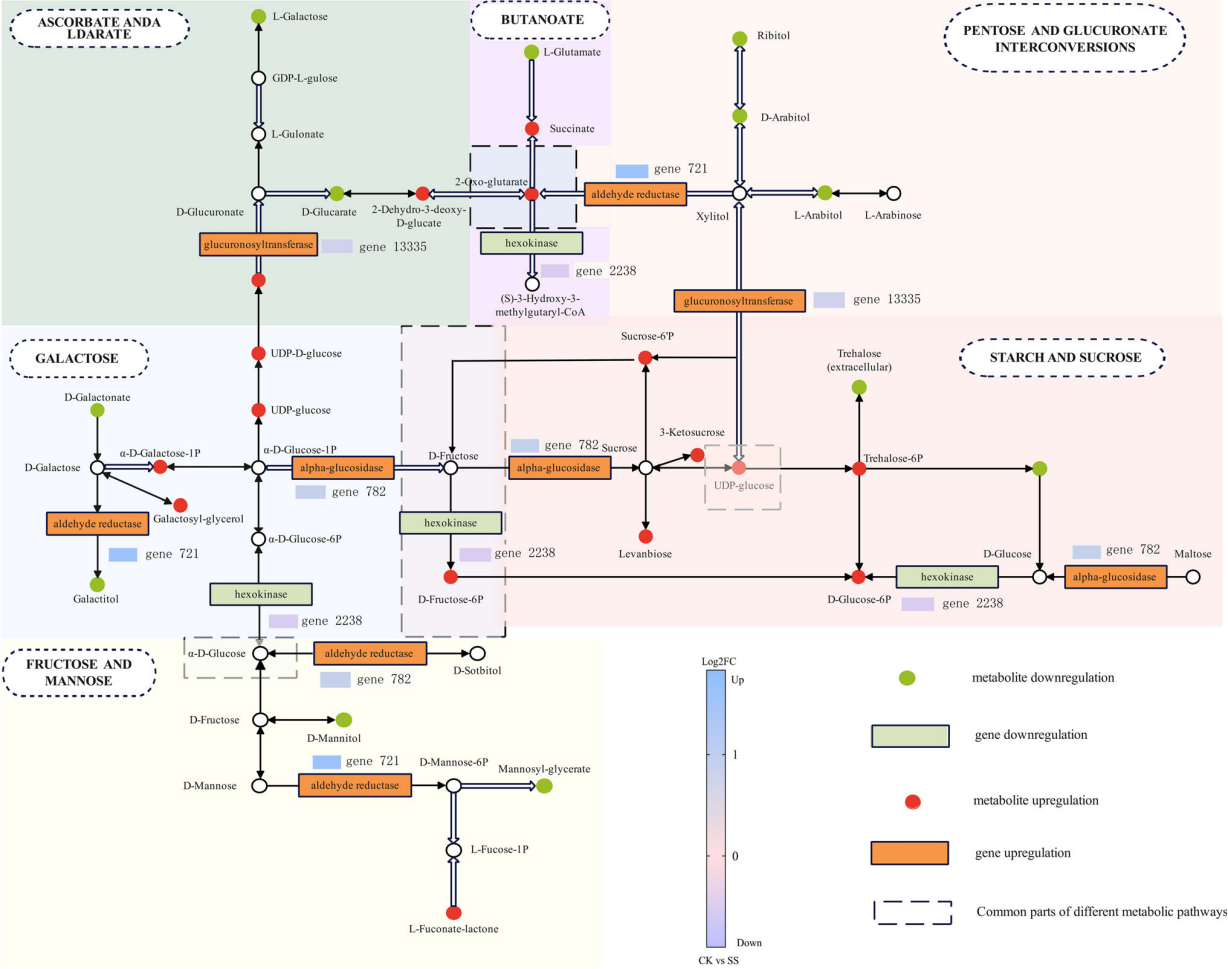
The co‐expression analysis of structural genes and metabolites within the sugar metabolism pathway.

To further elucidate the response of *Hippodamia variegata* to starvation stress, we integrated DEGs and DAMs into KEGG pathway analyses. KEGG enrichment analysis comparing the control and starvation groups revealed that 41 pathways were co‐enriched (Fig. S4(A)). The most significantly enriched pathways included starch and sucrose metabolism, fructose and mannose metabolism, galactose metabolism, ascorbic acid and aldose acid metabolism, and pentose and glucuronic acid metabolism. In addition, fatty acid synthesis, glyceride metabolism, and glycine, serine, and threonine metabolic pathways were enriched (Fig. S4(B)). Within the starch and sucrose metabolic pathway, two DEGs were enriched, one up‐regulated (*gene 782*) and one down‐regulated (*gene 2238*). Similarly, two DEGs were enriched in the fructose and mannose metabolic pathways, with two up‐regulated (*gene 721* and *gene 782*). The galactose metabolic pathway contained three enriched DEGs, two of which were up‐regulated (*gene 721* and *gene 782*) and one down‐regulated (*gene 2238*). In the metabolic pathways of ascorbic acid and glucuronic acid, only one DEG was up‐regulated, while the mutual conversion pathway of pentose and glucuronic acid showed two up‐regulated DEGs (Fig. 3).

### 3.6 Integrated WGCNA and key genes co‐expression network analysis

**Figure 4:**
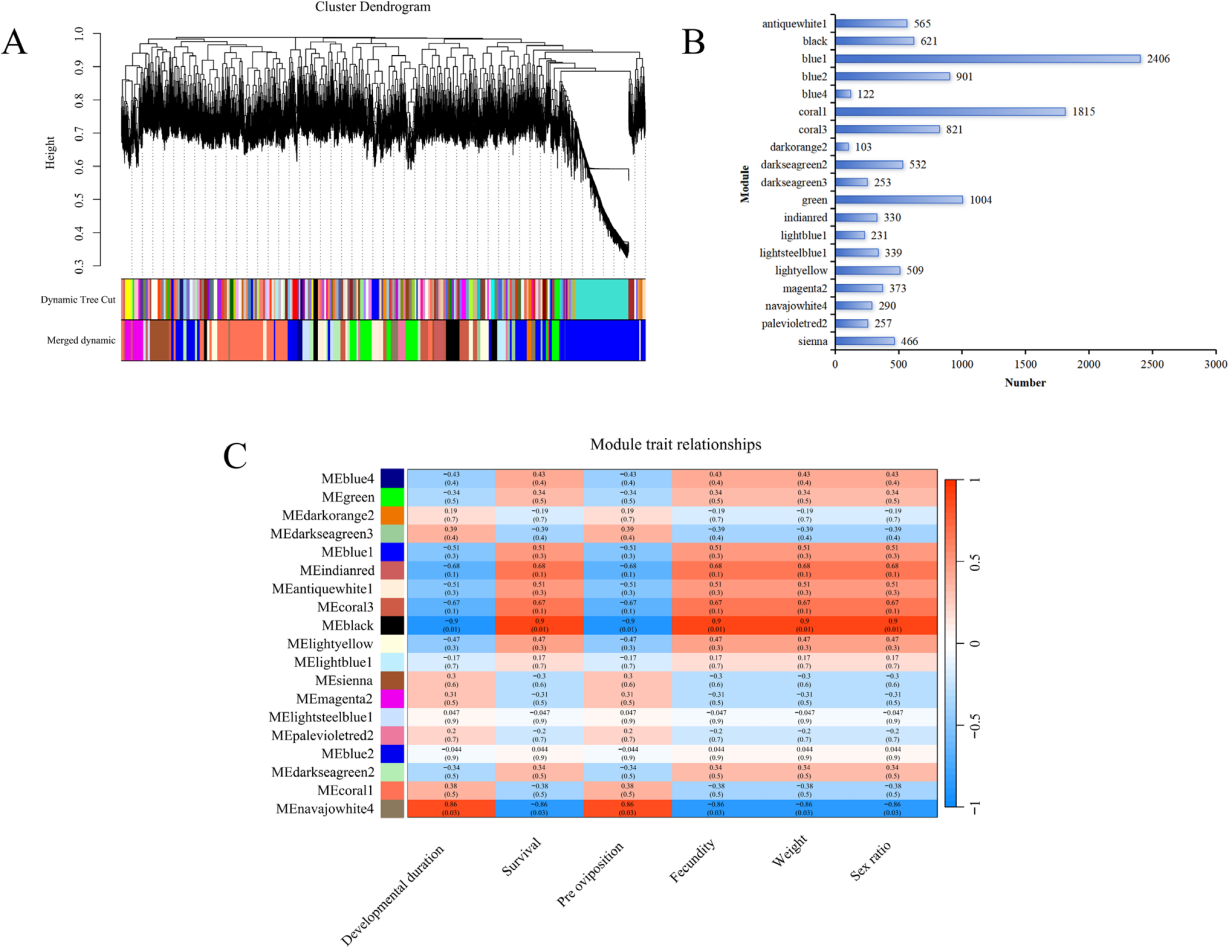
Weighted gene co‐expression network analysis (WGCNA) and differentially expressed genes (DEGs)‐phenotype correlation analysis of *Hippodamia variegata* under starvation stress. (A) Clustering dendrogram of genes and module division. (B) Module statistics bar chart. (C) Heat map of correlation between modules and traits.

Based on RNA‐sequencing data, WGCNA was employed to analyze the correlations between DEGs under starvation stress and developmental parameters, such as developmental duration, survival rate, and body weight. Different branches and colors in the cluster dendrogram represent distinct co‐expression patterns, with highly correlated genes clustered into the same module. The analysis revealed that a total of 11 938 genes were integrated into 19 modules, among which the blue1 module contained the largest number of genes (2406), while the darkorange2 module contained the fewest (103) (Fig. 4(A),(B)). The MEblack module exhibited the strongest correlation with various developmental parameters (correlation coefficient = 0.90, *P* = 0.01, Fig. 4(C) and Table S2). By combining the significantly enriched metabolic pathways identified in Section 3.5 and the DEGs of each pathway, it was found that gene 721 (*AKR1B1*, aldehyde reductase) was involved in multiple pathways related to carbohydrate and energy metabolism and clustered in the MEblack module. Therefore, we speculated that this gene may be involved in the response to starvation stress and selected it for functional verification.

### 3.7 Sequence analysis of the *HvarAKR1B1*1 gene in *Hippodamia variegata*

Based on the earlier findings, the *HvarAKR1B1* gene was screened. The full‐length ORF sequence of *HvarAKR1B1* was 948 bp, which was predicted to encode a protein of 315 amino acids. The predicted isoelectric point and molecular weight were calculated as 5.67 and 35.72 kDa, respectively. Conserved domain prediction revealed that the *HvarAKR1B1* protein of *Hippodamia variegata* shares a conserved TIM barrel structure with AKR1B1 proteins from other species. This structure is composed of ten parallel β‐folded plates surrounding a barrel core, which is further surrounded by ten α‐helical plates on the outside, creating a barrel‐like structure. The β‐folded sheet is located inside, and the α‐helix is located outside (Fig. S5(A)). Phylogenetic analysis showed that *HvarAKR1B1* is closely related to *Harmonia axyridis* and *Coccinella septempunctata*, with all three species belonging to the order Coleoptera (Fig. S5(B)).

### 3.8 Expression patterns of *HvarAKR1B1*

RT‐qPCR analysis showed that *HvarAKR1B1* is highly expressed in the fourth instar larvae, while those from egg to third instar larvae are the lowest (Fig. S6(A)). Tissue‐specific expression profiling indicated that the thorax displayed the highest expression level. In addition, the expression level of *HvarAKR1B1* in female adults is higher than that in males in both thorax and abdominal tissues. However, the expression levels in the feet and wings in males are higher than those in females (Fig. S6(B)).

### 3.9 Effects of silencing *HvarAKR1B1* on the fitness of *Hippodamia variegata*

**Figure 5:**
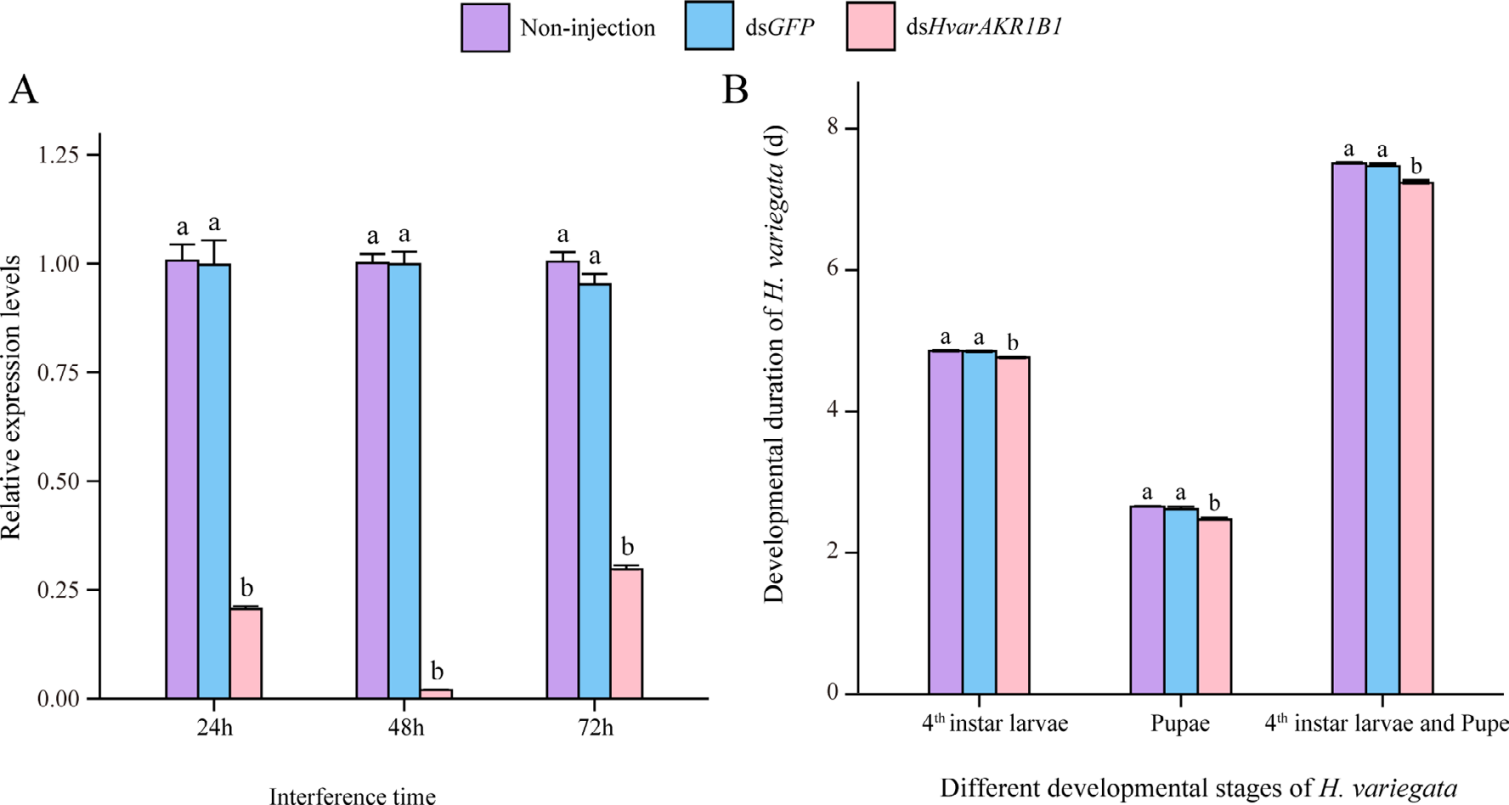
The efficiency of RNA interference of the *HvarAKR1B1* gene and its effect on the developmental duration of *Hippodamia variegata*. (A) The relative expression level of the *HvarAKR1B1* gene after interference; (B) The effect of silencing the *HvarAKR1B1* gene on the development period of *Hippodamia variegata*. Data in the figure are presented as mean ± standard error. Tukey's honestly significant difference (HSD) test was used for statistical analysis. The different lowercase letters indicated that there was a significant difference in the relative expression of the *HvarAKR1B1* gene between different treatments (*P* < 0.05).

The ds*HvarAKR1B1* was microinjected into the fourth instar larvae of *Hippodamia variegata*. Successful interference of the target gene was achieved within 24, 48, and 72 h. The expression level of *HvarAKR1B1* was significantly lower than that of the non‐injection and ds*GFP* groups after treating with the ds*HvarAKR1B1* for 24 h (*F* = 224.628, df = 2, *P* < 0.001), 48 h (*F* = 3360.294, df = 2, *P* < 0.001) and 72 h (*F* = 376.961, df = 2, *P* < 0.001), and the interference efficiency was 79.55%, 98.03% and 70.44%, respectively (Fig. 5(A)). In comparison with the non‐injection and ds*GFP* groups, the developmental duration of fourth instar larvae (*F* = 593.372, df = 2, *P* < 0.001), pupae (*F* = 12.382, df = 2, *P* = 0.007), and adults (*F* = 11.076, df = 2, *P* = 0.01) of *Hippodamia variegata* were significantly decreased after silencing *HvarAKR1B1* gene (Fig. 5(B)). Simultaneously, after silencing the *HvarAKR1B1* gene, the survival rate of fourth instar larvae (*F* = 37.155, df = 2, *P* < 0.001), emergence rate (*F* = 12.736, df = 2, *P* = 0.007) and the survival rate from the fourth instar larvae to adults (*F* = 48.408, df = 2, *P* < 0.001) of *Hippodamia variegata* were significantly lower than those of the non‐injected and ds*GFP* groups (Table 1).

**Table 1:** Effect of *HvarAKR1B1* gene silencing on the survival rate and initial eclosion weight of *Hippodamia variegata*

Treatment	Survival rate of fourth instar larvae	Emergence rate (%)	Survival rate from fourth instar larvae to adults (%)	Initial eclosion weight (mg)
ds*HvarAKR1B1*	84.84 ± 0.68b	89.51 ± 0.82b	75.94 ± 1.00b	4.68 ± 0.01a
ds*GFP*	93.33 ± 0.13a	92.85 ± 0.15a	86.66 ± 0.26a	4.67 ± 0.00a
Non‐injection	91.40 ± 1.06a	91.89 ± 0.11a	83.98 ± 0.93a	4.67 ± 0.00a

*Note*: Different lowercase letters in each column indicate significant differences in the corresponding indicators of *Hippodamia variegata* after the injection of ds*HvarAKR1B1*.

## 4 DISCUSSION

Starvation stress, an environmental challenge faced by organisms, exerts a wide range of physiological effects.^39^, ^40^ Compared with the normal reared *Hippodamia variegata*, the developmental duration of each developmental stage of *Hippodamia variegata* larvae under starvation stress was prolonged, and the survival rates of the first, second, and fourth instar larvae were significantly reduced. In addition, starvation stress significantly shortened the pupal maintenance period of *Hippodamia variegata*. A similar phenomenon was observed in *Helicoverpa armigera*, where the pupal stage was significantly shortened by 1.72 days after 3 days of starvation.^41^ The adaptive response to larval nutritional restriction also plays an important role in determining the adaptability of adults.^42^ This study further revealed that starvation led to a slowdown in ovarian development, a significant decrease in egg production, and a prolonged pre‐oviposition period, along with a population sex ratio biased towards males. The ovary functions not only as a reproductive organ but also as an energy reserve system.^43^, ^44^ Under nutrient deprivation, insects can mobilize energy through egg resorption,^45^ which may represent a physiological mechanism for reduced reproductive investment. The observed prolongation of the pre‐oviposition period and sex ratio shift align with the findings reported by Agarwala *et al*.^46^ These results collectively indicate that starvation stress significantly reduces the population fitness of *Hippodamia variegata*. To cope with such pressures, *Hippodamia variegata* may exhibit ecological adaptability and life history plasticity under adverse conditions by adjusting developmental rates, altering reproductive input, and adjusting sex ratio distributions.

Insect growth and development are regulated by a series of complex physiological and biochemical processes.^47^, ^48^ To systematically analyze the effect of starvation stress on these processes, omics technology, especially the integrated analysis of transcriptomic and metabolomic data, provides a powerful research paradigm.^41^, ^49^, ^50^ In this study, integrated transcriptomics and metabolomics analyses revealed that DEGs and DAMs were co‐enriched in glucose metabolism, lipid metabolism, and amino acid metabolism pathways, revealing the synergistic regulation network of *Hippodamia variegata* in response to starvation stress. In terms of glucose metabolism, starvation resulted in a significant decrease in trehalose and sucrose levels, while the content of trehalose‐6‐phosphate, an intermediate product of trehalose decomposition, increased, and the expression of the key enzyme *α*‐glucosidase‐encoding gene for sucrose decomposition was significantly up‐regulated. These findings align with the observations of decreased glycogen content in starved silkworms,^51^ suggesting that insects may respond to energy shortages by mobilizing glycogen reserves, activating glycolysis and utilizing carbohydrate intermediates.^22^, ^29^ As the main blood sugar in insects, trehalose is rapidly decomposed for energy supply when energy is scarce.^52^, ^53^ In addition, the up‐regulation of galactosidase and d‐fructose‐6‐phosphatase genes, as well as the decrease in mannose galactose‐related gene expression and metabolite content, indicates that starvation may promote the decomposition of structural sugars (e.g., mannose and galactose) to optimize energy allocation. A similar pattern was observed in *Helicoverpa armigera*, and the triglyceride, glycogen, and hemolymph trehalose levels in the starved larvae were significantly reduced.^54^ In terms of lipid metabolism, the expression of glycerol‐3‐phosphate dehydrogenase (GPDH) and the content of its product sn‐glycerol‐3‐phosphate increased significantly under starvation stress, indicating that lipid catabolism was enhanced. Lipids are an important energy reserve for insects and can be broken down to meet energy demands during starvation.^55^ GPDH, a key enzyme in glycerol metabolism, catalyzes the conversion of glycerol‐3‐phosphate to dihydroxyacetone phosphate, which enters the glycolytic pathway to further generate energy.^56^, ^57^ Regarding amino acid metabolism, the levels of several amino acids, including serine, leucine, asparagine, glutamate, and phenylalanine, were significantly reduced. We thus speculate that, in addition to catabolizing carbohydrates and lipids, *Hippodamia variegata* may also reduce amino acid synthesis or enhance their degradation to generate energy, thereby sustaining survival and mitigating the fitness costs imposed by starvation stress.

WGCNA showed that the MEblack module was significantly correlated with *Hippodamia variegata* suitability‐related parameters. In addition, most genes within this module may be related to the transport and metabolism of sugars, carbohydrates, and amino acid metabolites. By combining the WGCNA results and DAMs, it was found that 721 (*AKR1B1*, *aldehyde reductase*) is involved in multiple carbohydrate and energy metabolism‐related pathways and significantly up‐regulated. Aldosterone reductase (AKR), a group of NADPH‐dependent oxidoreductases, possesses a highly conserved three‐dimensional conformation of α/β‐barrel structure.^58^, ^59^ Members of the AKR superfamily are widely distributed across plants, animals, and prokaryotes, and are classified into 15 families and numerous subfamilies.^60^ Currently, research on the AKR family in humans is relatively extensive. It is highly up‐regulated in inflammatory diseases and various cancers and has become a biomarker for hepatocellular carcinoma, colorectal cancer, and non‐small cell lung cancer, among others.^61^, ^62^ In contrast, the identification and characterization of AKR family genes in insects remain limited. For instance, a 3DE 3β‐reductase was purified from the haemolymph of *Spodoptera littoralis*, and was identified as an aldol reductase based on amino acid homology. This protein is distributed across multiple tissues of the coastal armyworm and may be involved in regulating ecdysone concentrations.^63^ In *B. mori*, AKR2E8 is highly expressed in the midgut and may participate in detoxifying aldehydes present in mulberry leaves.^64^ Another novel gene, AKR2E4, was found to be up‐regulated following exposure to the pesticide diazinon, and it exhibits strong activity against the substrate inhibitor 3‐dehydroecdysone, indicating its potential involvement not only in detoxification processes but also in regulating ecdysone biosynthesis.^65^

RNAi has been widely adopted in entomological research as a powerful tool for studying gene function because of its high specificity, technical simplicity, and ability to suppress gene expression.^66^ Among various delivery approaches, microinjection stands out for its direct and precise application, enabling efficient RNAi‐mediated knockdown.^67^, ^68^, ^69^ For example, in *Hippodamia variegata*, injection of ds*HvarOREO* for 24 h or ds*HvarOBP5* for 72 h resulted in gene silencing efficiencies exceeding 95%.^8^ The results of this study showed that after injection of 1000 ng ds*HvarAKR1B1*, the interference efficiencies were 79.55%, 98.03%, and 70.44% at 24, 48, and 72 h, respectively, indicating that microinjection effectively silences this gene. In addition, RNAi‐mediated suppression of *HvarAKR1B1* significantly shortened the developmental duration of the fourth instar larvae, pupal stage, and total period from the fourth instar larvae to adult emergence. Concurrently, the adult emergence rate, eclosion rate, and survival to adulthood were significantly reduced. Beyond its role in aldehyde and ketone detoxification, *HvarAKR1B1* has been implicated in diapause‐related biochemical pathways, which are influenced by fluctuations in ecdysone or larval hormone titers.^70^ Therefore, we speculate that the up‐regulation of *HvarAKR1B1* may be related to the biochemical pathway of diapause, and its up‐regulated expression could be necessary to activate ecdysone synthesis.^71^ However, this speculation requires further study.

## 5 CONCLUSION

This study revealed the molecular mechanisms underlying the response of *Hippodamia variegata* to starvation stress at both the transcriptional and metabolic levels. The changes in carbohydrates, lipids, amino acids, and other energy‐related substances under starvation stress were found to affect the growth, development, and fecundity of *Hippodamia variegata*. In addition, the role of *HvarAKR1B1* in mediating starvation resistance was investigated using RNAi. Our findings not only highlight the key role of *HvarAKR1B1* in starvation tolerance but also offer insights into the adaptation mechanisms and survival strategies of *Hippodamia variegata* under different environmental conditions and provide a more comprehensive theoretical basis for the effective utilization of this species in biological control.

## CONFLICT OF INTEREST

The authors declare no competing financial interests.

## Supporting information


**Table S1.** Primer information.
**Table S2.** Correlation coefficient between modules and traits.
**Figure S1.** Analysis of DEGs in *Hippodamia variegata* under starvation stress. (A) The number of DEGs between the starvation stress and the control groups. (B) KEGG enrichment analysis of DEGs between the starvation stress and the control groups. The *X*‐axis shows the enrichment factor. The sizes of the circle indicate the total number of enriched genes.
**Figure S2.** GO functional annotations of DEGs of *Hippodamia variegata* in the comparison between starvation stress and the control group. (A–C) classified by cellular components (CCs), biological processes (BPs) and molecular functions (MFs).
**Figure S3.** Metabolomic analysis of *Hippodamia variegata* under starvation conditions. (A) DAMs of *Hippodamia variegata* between the starvation stress and the control groups. (B) The quantity of differential metabolites between the starvation stress and the control group. (C) KEGG enrichment analysis of DAMs between the starvation stress and the control group.
**Figure S4.** Integrated analysis of DAMs and DEGs of *Hippodamia variegata*. (A) Venn diagram showing the number of common and unique pathways in the comparison between starvation stress and the control group. (B) Common pathway showing the degree of enrichment of DAMs in the comparison between starvation stress and the control group.
**Figure S5.** Bioinformatics and phylogenetic analysis of AKR1B1. (A) Secondary structure prediction of AKR1B1 of *Hippodamia variegata*. *β*1–*β*10: *β*‐folded sheet; *α*1–*α*10: *α* helix; *η*1–*η*5: disulfide bond. (B) Phylogenetic analysis of *Hippodamia variegata* with other AKR1B1 sequences from other species. The phylogenetic tree is based on amino acid sequences using the neighbor‐joining method with a bootstrap of 1000 through MEGA11.0.
**Figure S6.** Relative expression level of *HvarAKR1B1*. (A) Across different developmental stages of *Hippodamia variegata*. (B) In different tissues of *Hippodamia variegata*. Asterisks indicate significant differences between females and males (ns, not significant, *P < 0.05, **P < 0.01, ***P < 0.001, *t*‐test). Different lowercase letters indicate significant differences among different developmental stages (one‐way ANOVA).

## Data Availability

The data that support the findings of this study are available from the corresponding author upon reasonable request.

## References

[1] Carletto J , Martin T , Vanlerberghe‐Masutti F and Brévault T , Insecticide resistance traits differ among and within host races in *Aphis gossypii* . Pest Manag Sci 66:301–307 (2010). 19908228 10.1002/ps.1874

[2] Ebert TA and Cartwright BO , Biology and ecology of *Aphis gossypii* glover (Homoptera: Aphididae). Southwest Entomol 22:116–153 (1997).

[3] Pan Y , Wen S , Chen X , Gao X , Zeng X , Liu X *et al*., UDP‐glycosyltransferases contribute to spirotetramat resistance in *Aphis gossypii* glover. Pestic Biochem Physiol 166:104565 (2020). 32448419 10.1016/j.pestbp.2020.104565

[4] Wei ZH , Zhao P , Ning XY , Xie YQ , Li Z and Liu XX , Nanomaterial‐encapsulated dsRNA‐targeting chitin pathway‐a potential efficient and eco‐friendly strategy against cotton aphid, *Aphis gossypii* (Hemiptera: Aphididae). J Agric Food Chem 72:20905–20917 (2024). 39258562 10.1021/acs.jafc.4c06390

[5] Mahas JW , Steury TD , Huseth AS and Jacobson AL , Imidacloprid‐resistant *Aphis gossypii* populations are more common in cotton‐dominated landscapes. Pest Manag Sci 79:1040–1047 (2023). 36327354 10.1002/ps.7274

[6] Zhang L , Wei Y , Wei L , Liu X and Liu N , Effects of transgenic cotton lines expressing dsAgCYP6CY3‐P1 on the growth and detoxification ability of *Aphis gossypii* glover. Pest Manag Sci 79:481–488 (2023). 36196669 10.1002/ps.7220

[7] Liu TX and Chen XX , Biological control of *aphids* in China: successes and prospects. Annu Rev Entomol 70:401–419 (2025). 39499910 10.1146/annurev-ento-121423-012130

[8] Tang H , Xie J , Liu J , Khashaveh A , Liu X , Yi C *et al*., Odorant‐binding protein HvarOBP5 in ladybird *Hippodamia variegata* regulates the perception of semiochemicals from preys and habitat plants. J Agric Food Chem 71:1067–1076 (2023). 36598383 10.1021/acs.jafc.2c07355

[9] Zarei M , Madadi H , Zamani AA and Nedvěd O , Intraguild predation between *Chrysoperla carnea* (Neuroptera: Chrysopidae) and *Hippodamia variegata* (Coleoptera: Coccinellidae) at various extraguild prey densities and arena complexities. Insects 11:288 (2020). 32397273 10.3390/insects11050288 PMC7291017

[10] Halsch CA , Shapiro AM , Fordyce JA , Nice CC , Thorne JH , Waetjen DP *et al*., Insects and recent climate change. Proc Natl Acad Sci USA 118:e2002543117 (2021). 33431560 10.1073/pnas.2002543117 PMC7812774

[11] Yan J , Kim CH , Chesser L , Ramirez JL and Stone CM , Nutritional stress compromises mosquito fitness and antiviral immunity, while enhancing dengue virus infection susceptibility. Commun Biol 6:1123 (2023). 37932414 10.1038/s42003-023-05516-4 PMC10628303

[12] Zhao Z , Zhang X , Zhao F , Zhou Z , Zhao F , Wang J *et al*., Stress responses of the intestinal digestion, antioxidant status, microbiota and non‐specific immunity in Songpu mirror carp (*Cyprinus carpio* L.) under starvation. Fish Shellfish Immunol 120:411–420 (2022). 34915148 10.1016/j.fsi.2021.12.008

[13] Carrión PJA , Desai N , Brennan JJ , Fifer JE , Siggers T , Davies SW *et al*., Starvation decreases immunity and immune regulatory factor NF‐*κ*B in the starlet sea anemone *Nematostella vectensis* . Commun Biol 6:698 (2023). 37420095 10.1038/s42003-023-05084-7 PMC10329013

[14] Truman JW , The evolution of insect metamorphosis. Curr Biol 29:1252–1268 (2019). 10.1016/j.cub.2019.10.009 31794762

[15] Awde DN , Řeřicha M and Knapp M , Increased pupal temperature has reversible effects on thermal performance and irreversible effects on immune system and fecundity in adult ladybirds. Commun Biol 6:838 (2023). 37573399 10.1038/s42003-023-05196-0 PMC10423239

[16] Ovchinnikov A , Reznik S , Bezman‐Moseyko O and Belyakova N , Walking activity of a predatory ladybird, *Cheilomenes propinqua*: impacts of photoperiod, temperature, and starvation. BioControl 67:513–522 (2022).

[17] Sánchez‐Paz A , García‐Carreño F , Muhlia‐Almazán A , Peregrino‐Uriarte AB , Hernández‐López J and Yepiz‐Plascencia G , Usage of energy reserves in crustaceans during starvation: status and future directions. Insect Biochem Mol Biol 36:241–249 (2006). 16551538 10.1016/j.ibmb.2006.01.002

[18] Enriquez T and Visser B , The importance of fat accumulation and reserves for insect overwintering. Curr Opin Insect Sci 60:101118 (2023). 37739063 10.1016/j.cois.2023.101118

[19] Helm BR , Rinehart JP , Yocum GD , Greenlee KJ and Bowsher JH , Metamorphosis is induced by food absence rather than a critical weight in the solitary bee, *Osmia lignaria* . Proc Natl Acad Sci USA 114:10924–10929 (2017). 28973885 10.1073/pnas.1703008114 PMC5642682

[20] Fischer N , Costa CP , Hur M , Kirkwood JS and Woodard SH , Impacts of neonicotinoid insecticides on bumble bee energy metabolism are revealed under nectar starvation. Sci Total Environ 912:169388 (2024). 38104805 10.1016/j.scitotenv.2023.169388

[21] Kubrak O , Koyama T , Ahrentløv N , Jensen L , Malita A , Naseem MT *et al*., The gut hormone Allatostatin C/somatostatin regulates food intake and metabolic homeostasis under nutrient stress. Nat Commun 13:692 (2022). 35121731 10.1038/s41467-022-28268-x PMC8816919

[22] Zhang DW , Xiao ZJ , Zeng BP , Li K and Tang YL , Insect behavior and physiological adaptation mechanisms under starvation stress. Front Physiol 10:163 (2019). 30890949 10.3389/fphys.2019.00163 PMC6411660

[23] Shi ZK , Wang S , Wang SG , Zhang L , Xu YX , Guo XJ *et al*., Effects of starvation on the carbohydrate metabolism in *Harmonia axyridis* (Pallas). Biol Open 6:1096–1103 (2017). 28606937 10.1242/bio.025189 PMC5550912

[24] Brückner A and Heethoff M , Fatty acid metabolism in an oribatid mite: de novo biosynthesis and the effect of starvation. Exp Appl Acarol 81:483–494 (2020). 32748182 10.1007/s10493-020-00529-8

[25] Raushenbach IY , Gruntenko NE , Bownes M , Adonieva NV , Terashima J , Karpova EK *et al*., The role of juvenile hormone in the control of reproductive function in *Drosophila virilis* under nutritional stress. J Insect Physiol 50:323–330 (2004). 15081825 10.1016/j.jinsphys.2004.02.001

[26] Li Z , Ma R , Wang L , Wang Y , Qin Q , Chen L *et al*., Starvation stress affects iron metabolism in honeybee *Apis mellifera* . Biologia 77:2133–2148 (2022).

[27] Nakajima E , Shimaji K , Umegawachi T , Tomida S , Yoshida H , Yoshimoto N *et al*., The histone deacetylase gene Rpd3 is required for starvation stress resistance. PLoS One 11:e0167554 (2016). 27907135 10.1371/journal.pone.0167554 PMC5132236

[28] Zhou J , Chen X , Yan J , You K , Yuan Z , Zhou Q *et al*., Brummer‐dependent lipid mobilization regulates starvation resistance in *Nilaparvata lugens* . Arch Insect Biochem Physiol 99:e21481 (2018). 29956367 10.1002/arch.21481

[29] Dai TM , Qiu JF , Luo C , Cui WZ , Liu K , Li JL *et al*., The circadian clock affects starvation resistance through the pentose phosphate pathway in silkworm, Bombyx mori. Insect Sci 32:55–68 (2025). 38769889 10.1111/1744-7917.13381

[30] Hu D , Luo W , Fan LF , Liu FL , Gu J , Deng HM *et al*., Dynamics and regulation of glycolysis‐tricarboxylic acid metabolism in the midgut of *Spodoptera litura* during metamorphosis. Insect Mol Biol 25:153–162 (2016). 26683413 10.1111/imb.12208

[31] Wang W , Yao J and Li HB , Predatory functional responses of *Aphis gossypii* by three ladybugs. Chin J Biol Control 24:15–20 (2008).

[32] Andrews S and FastQC , A quality control tool for high throughput sequence data (2014).

[33] Kim D , Paggi JM , Park C , Bennett C and Salzberg SL , Graph‐based genome alignment and genotyping with HISAT2 and HISAT‐genotype. Nat Biotechnol 37:907–915 (2019). 31375807 10.1038/s41587-019-0201-4 PMC7605509

[34] Robinson MD , McCarthy DJ and Smyth GK , edgeR: a Bioconductor package for differential expression analysis of digital gene expression data. Bioinformatics 26:139–140 (2010). 19910308 10.1093/bioinformatics/btp616 PMC2796818

[35] Xu S , Hu E , Cai Y , Xie Z , Luo X , Zhan L *et al*., Using clusterProfiler to characterize multiomics data. Nat Protoc 19:3292–3320 (2024). 39019974 10.1038/s41596-024-01020-z

[36] Langfelder P and Horvath S , WGCNA: an R package for weighted correlation network analysis. BMC Bioinformatics 9:559 (2008). 19114008 10.1186/1471-2105-9-559 PMC2631488

[37] Livak KJ and Schmittgen TD , Analysis of relative gene expression data using real‐time quantitative PCR and the 2(‐Delta Delta C(T)) method. Methods 25:402–408 (2001). 11846609 10.1006/meth.2001.1262

[38] Xie J , Liu T , Khashaveh A , Yi C , Liu X and Zhang Y , Identification and evaluation of suitable reference genes for RT‐qPCR analysis in *Hippodamia variegata* (Coleoptera: Coccinellidae) under different biotic and abiotic conditions. Front Physiol 12:669510 (2021). 34079474 10.3389/fphys.2021.669510 PMC8165390

[39] Fan X , Hou T , Sun T , Zhu L , Zhang S , Tang K *et al*., Starvation stress affects the maternal development and larval fitness in zebrafish (*Danio rerio*). Sci Total Environ 695:133897 (2019). 31425978 10.1016/j.scitotenv.2019.133897

[40] Teets NM , Marshall KE and Reynolds JA , Molecular mechanisms of winter survival. Annu Rev Entomol 68:319–339 (2023). 36206770 10.1146/annurev-ento-120120-095233

[41] Liu Y , Wang X , Luo S , Ma L , Zhang W , Xuan S *et al*., Metabolomic and transcriptomic analyses identify quinic acid protecting eggplant from damage caused by western flower thrips. Pest Manag Sci 78:5113–5123 (2022). 36053852 10.1002/ps.7129

[42] Tigreros N , Linking nutrition and sexual selection across life stages in a model butterfly system. Funct Ecol 27:145–154 (2013).

[43] Jin J and Zhao T , Niche formation and function in developing tissue: studies from the *Drosophila ovary* . Cell Commun Signal 21:23 (2023). 36707894 10.1186/s12964-022-01035-7 PMC9881360

[44] Lynch JA , Evolution of maternal control of axial patterning in insects. Curr Opin Insect Sci 31:37–42 (2019). 31109671 10.1016/j.cois.2018.07.011

[45] Ferrer A , Dixon AFG , Gibernau M and Hemptinne JL , Ovarian dynamics and specialisation in ladybirds. Ecol Entomol 35:100–103 (2010).

[46] Agarwala BK , Yasuda H and Sato S , Life history response of a predatory ladybird, *Harmonia axyridis* (Pallas) (Coleoptera: Coccinellidae), to food stress. Appl Entomol Zool 43:183–189 (2008).

[47] Gu X , Jouandin P , Lalgudi PV , Binari R , Valenstein ML , Reid MA *et al*., Sestrin mediates detection of and adaptation to low‐leucine diets in *Drosophila* . Nature 608:209–216 (2022). 35859173 10.1038/s41586-022-04960-2 PMC10112710

[48] Kosakamoto H , Sakuma C , Okada R , Miura M and Obata F , Context‐dependent impact of the dietary non‐essential amino acid tyrosine on *Drosophila* physiology and longevity. Sci Adv 10:eadn7167 (2024). 39213345 10.1126/sciadv.adn7167 PMC11364096

[49] Jiang W , Wu M , Fan J , Lu C , Dong W , Chen W *et al*., Integrated metabolomic and transcriptomic profiling reveals the defense response of tea plants (*Camellia sinensis*) to *Toxoptera aurantii* . J Agric Food Chem 72:27125–27138 (2024). 39579374 10.1021/acs.jafc.4c10093

[50] Li F , Yu Y , Guo M , Lin Y , Jiang Y , Qu M *et al*., Integrated analysis of physiological, transcriptomics and metabolomics provides insights into detoxication disruption of PFOA exposure in *Mytilus edulis* . Ecotoxicol Environ Saf 214:112081 (2021). 33677383 10.1016/j.ecoenv.2021.112081

[51] Satake S , Kawabe Y and Mizoguchi A , Carbohydrate metabolism during starvation in the silkworm *Bombyx mori* . Arch Insect Biochem Physiol 44:90–98 (2000). 10861869 10.1002/1520-6327(200006)44:2<90::AID-ARCH4>3.0.CO;2-0

[52] Mizutani K , Yoshida Y , Nakanishi E , Miyata Y , Tokumoto S , Fuse H *et al*., A sodium‐dependent trehalose transporter contributes to anhydrobiosis in insect cell line, Pv11. Proc Natl Acad Sci USA 121:e2317254121 (2024). 38551840 10.1073/pnas.2317254121 PMC10998604

[53] Wu H , Sun C , Wu Y , Cao C and Sun L , Physiological roles of trehalose in *Hyphantria cunea* revealed by RNA interference of trehalose‐6‐phosphate synthase and trehalase genes. Pestic Biochem Physiol 211:106422 (2025). 40350235 10.1016/j.pestbp.2025.106422

[54] Jiang T , Ma L , Liu XY , Xiao HJ and Zhang WN , Effects of starvation on respiratory metabolism and energy metabolism in the cotton bollworm *Helicoverpa armigera* (Hübner) (Lepidoptera: Noctuidae). J Insect Physiol 119:103951 (2019). 31563619 10.1016/j.jinsphys.2019.103951

[55] Toprak U , The role of peptide hormones in insect lipid metabolism. Front Physiol 11:434 (2020). 32457651 10.3389/fphys.2020.00434 PMC7221030

[56] Oakeshott JG , Gibson JB , Anderson PR , Knibb WR , Anderson DG and Chambers GK , Alcohol dehydrogenase and glycerol‐3‐phosphate dehydrogenase clines in *Drosophila melanogaster* on different continents. Evolution 36:86–96 (1982). 28581103 10.1111/j.1558-5646.1982.tb05013.x

[57] Oh S , Mai XL , Kim J , de Guzman ACV , Lee JY and Park S , Glycerol 3‐phosphate dehydrogenases (1 and 2) in cancer and other diseases. Exp Mol Med 56:1066–1079 (2024). 38689091 10.1038/s12276-024-01222-1 PMC11148179

[58] Maccari R and Ottanà R , In search for inhibitors of human aldo‐keto reductase 1B10 (AKR1B10) as novel agents to fight cancer and chemoresistance: current state‐of‐the‐art and prospects. J Med Chem 68:860–885 (2025). 39757466 10.1021/acs.jmedchem.4c01116

[59] Penning TM , The aldo‐keto reductases (AKRs): overview. Chem Biol Interact 234:236–246 (2015). 25304492 10.1016/j.cbi.2014.09.024 PMC4388799

[60] Zhao Q , Han B , Wang L , Wu J , Wang S , Ren Z *et al*., AKR1B1‐dependent fructose metabolism enhances malignancy of cancer cells. Cell Death Differ 31:1611–1624 (2024). 39406918 10.1038/s41418-024-01393-4 PMC11618507

[61] Qu J , Li J , Zhang Y , He R , Liu X , Gong K *et al*., AKR1B10 promotes breast cancer cell proliferation and migration via the PI3K/AKT/NF‐*κ*B signaling pathway. Cell Biosci 11:163 (2021). 34419144 10.1186/s13578-021-00677-3 PMC8379827

[62] Shen Y , Qiu A , Huang X , Wen X , Shehzadi S , He Y *et al*., AKR1B10 and digestive tumors development: a review. Front Immunol 15:1462174 (2024). 39737179 10.3389/fimmu.2024.1462174 PMC11682995

[63] Takeuchi H , Chen JH , O'Reilly DR , Rees HH and Turner PC , Regulation of ecdysteroid signalling: molecular cloning, characterization and expression of 3‐dehydroecdysone 3 alpha‐reductase, a novel eukaryotic member of the short‐chain dehydrogenases/reductases superfamily from the cotton leafworm. Spodoptera littoralis Biochem J 349:239–245 (2000). 10861234 10.1042/0264-6021:3490239 PMC1221143

[64] Yamamoto K and Endo S , *Bombyx mori*‐derived aldo‐keto reductase AKR2E8 detoxifies aldehydes present in mulberry leaves. Chem Biol Interact 351:109717 (2022). 34737151 10.1016/j.cbi.2021.109717

[65] Yamamoto K and Endo S , Novel aldo‐keto reductase AKR2E9 regulates aldehyde content in the midgut and antennae of the silkworm (*Bombyx mori*). Arch Insect Biochem Physiol 112:e21979 (2023). 36283966 10.1002/arch.21979

[66] Zhu KY and Palli SR , Mechanisms, applications, and challenges of insect RNA interference. Annu Rev Entomol 65:293–311 (2020). 31610134 10.1146/annurev-ento-011019-025224 PMC9939233

[67] Cooper AM , Song H , Yu Z , Biondi M , Bai J , Shi X *et al*., Comparison of strategies for enhancing RNA interference efficiency in *Ostrinia nubilalis* . Pest Manag Sci 77:635–645 (2021). 33002336 10.1002/ps.6114 PMC7855606

[68] Dalaisón‐Fuentes LI , Pascual A , Crespo M , Andrada NL , Welchen E and Catalano MI , Knockdown of double‐stranded RNases (dsRNases) enhances oral RNA interference (RNAi) in the corn leafhopper, *Dalbulus maidis* . Pestic Biochem Physiol 196:105618 (2023). 37945254 10.1016/j.pestbp.2023.105618

[69] Zhang X , Fan Z , Wang Q , Kong X , Liu F , Fang J *et al*., RNAi efficiency through dsRNA injection is enhanced by knockdown of dsRNA nucleases in the fall webworm, *Hyphantria cunea* (Lepidoptera: Arctiidae). Int J Mol Sci 23:6182 (2022). 35682860 10.3390/ijms23116182 PMC9181381

[70] Kamphuis LG , Klingler JP , Jacques S , Gao LL , Edwards OR and Singh KB , Additive and epistatic interactions between AKR and AIN loci conferring bluegreen aphid resistance and hypersensitivity in *Medicago truncatula* . J Exp Bot 70:4887–4902 (2019). 31087095 10.1093/jxb/erz222 PMC6760273

[71] Reynolds JA and Hand SC , Embryonic diapause highlighted by differential expression of mRNAs for ecdysteroidogenesis, transcription and lipid sparing in the cricket *Allonemobius socius* . J Exp Biol 212:2075–2084 (2009). 19525434 10.1242/jeb.027367 PMC2702454

