# Supplementary material for: HvarAKR1B1 ‐mediated tolerance in Hippodamia variegata : Deciphering the metabolic adaptation and fitness costs under prolonged starvation
Source: Pest Manag Sci. 2026 Apr 23;82(8):7903–14. doi: 10.1002/ps.70851 (PMC13352252; doi:10.1002/ps.70851)
Supplement: Supplementary file 1 — Table S1. Primer information. Table S2. Correlation coefficient between modules and traits. Figure S1. Analysis of DEGs in Hippodamia variegata under starvation stress. (A) The number of DEGs between the starvation stress and the control groups. (B) KEGG enrichment analysis of DEGs between the starvation stress and the control groups. The X‐axis shows the enrichment factor. The sizes of the circle indicate the total number of enriched genes. Figure S2. GO functional annotations of DEGs of Hippodamia variegata in the comparison between starvation stress and the control group. (A–C) classified by cellular components (CCs), biological processes (BPs) and molecular functions (MFs). Figure S3. Metabolomic analysis of Hippodamia variegata under starvation conditions. (A) DAMs of Hippodamia variegata between the starvation stress and the control groups. (B) The quantity of differential metabolites between the starvation stress and the control group. (C) KEGG enrichment analysis of DAMs between the starvation stress and the control group. Figure S4. Integrated analysis of DAMs and DEGs of Hippodamia variegata. (A) Venn diagram showing the number of common and unique pathways in the comparison between starvation stress and the control group. (B) Common pathway showing the degree of enrichment of DAMs in the comparison between starvation stress and the control group. Figure S5. Bioinformatics and phylogenetic analysis of AKR1B1. (A) Secondary structure prediction of AKR1B1 of Hippodamia variegata. β1–β10: β‐folded sheet; α1–α10: α helix; η1–η5: disulfide bond. (B) Phylogenetic analysis of Hippodamia variegata with other AKR1B1 sequences from other species. The phylogenetic tree is based on amino acid sequences using the neighbor‐joining method with a bootstrap of 1000 through MEGA11.0. Figure S6. Relative expression level of HvarAKR1B1. (A) Across different developmental stages of Hippodamia variegata. (B) In different tissues of Hippodamia variegata. Asterisks indicate signi [file PS-82-7903-s001.docx]

**Table S1** Primer information

| **Application** | **Primer name** | **Primer sequence (5’-3’)** |
| --- | --- | --- |
| Clone | *HvarAKR1B1-F* | ATGATACCGAAAAAATTGAAA |
|  | *HvarAKR1B1-R* | TTAGTAATCATCGTGGAAAGG |
| RNAi | ds*HvarAKR1B1-F-1* | TAATACGACTCACTATAGGTTGAAGGATTCGCTGAGAA |
|  | ds*HvarAKR1B1-R-1* | TTCAGCCACTTTGGGGTCG |
|  | ds*GFP-F-1* | TAATACGACTCACTATAGGCCACAAGTTCAGCGTGTCCG |
|  | ds*GFP-R-1* | AAGTTCACCTTGATGCCGTTCT |
| RT-qPCR | *HvarAKR1B1-qF* | CCATCATAGGGCTTGGGACC |
|  | *HvarAKR1B1-qR* | CCAACCTCAACTTCATTTCCGT |
|  | EF1α-F | AGCCAACATTACCACTGA |
|  | EF1α-R | GTATCCACGACGCAATTC |

Note: The underline sequence represents the T7 promoter sequence introduced by the primer 5’.

**Table S2** Correlation coefficient between modules and traits

| **Modules** | **Developmental duration** | **Survival** | **Pre oviposition** | **Fecundity** | **Weight** | **Sex ratio** |
| --- | --- | --- | --- | --- | --- | --- |
| **MEblue4** | R^2^=-0.43, *p*=0.4 | R^2^=0.43, *p*=0.4 | R^2^=-0.43, *p*=0.4 | R^2^=0.43, *p*=0.4 | R^2^=0.43, *p*=0.4 | R^2^=0.43, *p*=0.4 |
| **MEgreen** | R^2^=-0.34, *p*=0.5 | R^2^=0.34, *p*=0.5 | R^2^=-0.34, *p*=0.5 | R^2^=-0.34, *p*=0.5 | R^2^=-0.34, *p*=0.5 | R^2^=-0.34, *p*=0.5 |
| **MEdarkorange2** | R^2^=0.19, *p*=0.7 | R^2^=-0.19, *p*=0.7 | R^2^=0.19, *p*=0.7 | R^2^=-0.19, *p*=0.7 | R^2^=-0.19, *p*=0.7 | R^2^=-0.19, *p*=0.7 |
| **MEdarseagreen3** | R^2^=0.39, *p*=0.4 | R^2^=-0.39, *p*=0.4 | R^2^=0.39, *p*=0.4 | R^2^=-0.39, *p*=0.4 | R^2^=-0.39, *p*=0.4 | R^2^=-0.39, *p*=0.4 |
| **MEblue1** | R^2^=-0.51, *p*=0.3 | R^2^=0.51, *p*=0.3 | R^2^=-0.51, *p*=0.3 | R^2^=-0.51, *p*=0.3 | R^2^=0.51, *p*=0.3 | R^2^=0.51, *p*=0.3 |
| **MEindianred** | R^2^=-0.68, *p*=0.1 | R^2^=0.68, *p*=0.1 | R^2^=-0.68, *p*=0.1 | R^2^=0.68, *p*=0.1 | R^2^=0.68, *p*=0.1 | R^2^=0.68, *p*=0.1 |
| **MEantiquewhite1** | R^2^=-0.51, *p*=0.3 | R^2^=0.51, *p*=0.3 | R^2^=-0.51, *p*=0.3 | R^2^=0.51, *p*=0.3 | R^2^=0.51, *p*=0.3 | R^2^=0.51, *p*=0.3 |
| **MEcoral3** | R^2^=-0.67, *p*=0.1 | R^2^=0.67, *p*=0.1 | R^2^=-0.67, *p*=0.1 | R^2^=0.67, *p*=0.1 | R^2^=0.67, *p*=0.1 | R^2^=0.67, *p*=0.1 |
| **MEblack** | R^2^=-0.9, *p*=0.01 | R^2^=0.9, *p*=0.01 | R^2^=-0.9, *p*=0.01 | R^2^=0.9, *p*=0.01 | R^2^=0.9, *p*=0.01 | R^2^=0.9, *p*=0.01 |
| **MElightyellow** | R^2^=-0.47, *p*=0.3 | R^2^=0.47, *p*=0.3 | R^2^=-0.47, *p*=0.3 | R^2^=0.47, *p*=0.3 | R^2^=0.47, *p*=0.3 | R^2^=0.47, *p*=0.3 |
| **MElightblue1** | R^2^=-0.17, *p*=0.7 | R^2^=0.17, *p*=0.7 | R^2^=-0.17, *p*=0.7 | R^2^=0.17, *p*=0.7 | R^2^=0.17, *p*=0.7 | R^2^=0.17, *p*=0.7 |
| **MEsienna** | R^2^=0.3, *p*=0.6 | R^2^=-0.3, *p*=0.6 | R^2^=0.3, *p*=0.6 | R^2^=-0.3, *p*=0.6 | R^2^=-0.3, *p*=0.6 | R^2^=-0.3, *p*=0.6 |
| **MEmagenta2** | R^2^=0.31, *p*=0.5 | R^2^=-0.31, *p*=0.5 | R^2^=0.31, *p*=0.5 | R^2^=-0.31, *p*=0.5 | R^2^=-0.31, *p*=0.5 | R^2^=-0.31, *p*=0.5 |
| **MElightsteelblue1** | R^2^=0.047, *p*=0.9 | R^2^=-0.047, *p*=0.9 | R^2^=0.047, *p*=0.9 | R^2^=-0.047, *p*=0.9 | R^2^=-0.047, *p*=0.9 | R^2^=-0.047, *p*=0.9 |
| **MEpalevioletred2** | R^2^=0.2, *p*=0.7 | R^2^=-0.2, *p*=0.7 | R^2^=0.2, *p*=0.7 | R^2^=-0.2, *p*=0.7 | R^2^=-0.2, *p*=0.7 | R^2^=-0.2, *p*=0.7 |
| **MEblue2** | R^2^=-0.044, *p*=0.9 | R^2^=0.044, *p*=0.9 | R^2^=-0.044, *p*=0.9 | R^2^=0.044, *p*=0.9 | R^2^=0.044, *p*=0.9 | R^2^=0.044, *p*=0.9 |
| **MEdarkseagreen2** | R^2^=-0.34, *p*=0.5 | R^2^=0.34, *p*=0.5 | R^2^=-0.34, *p*=0.5 | R^2^=0.34, *p*=0.5 | R^2^=0.34, *p*=0.5 | R^2^=0.34, *p*=0.5 |
| **MEcoral1** | R^2^=0.38, *p*=0.5 | R^2^=-0.38, *p*=0.5 | R^2^=0.38, *p*=0.5 | R^2^=-0.38, *p*=0.5 | R^2^=-0.38, *p*=0.5 | R^2^=-0.38, *p*=0.5 |
| **MEnavajowhite4** | R^2^=0.86, *p*=0.03 | R^2^=-0.86, *p*=0.03 | R^2^=0.86, *p*=0.03 | R^2^=-0.86, *p*=0.03 | R^2^=-0.86, *p*=0.03 | R^2^=-0.86, *p*=0.03 |


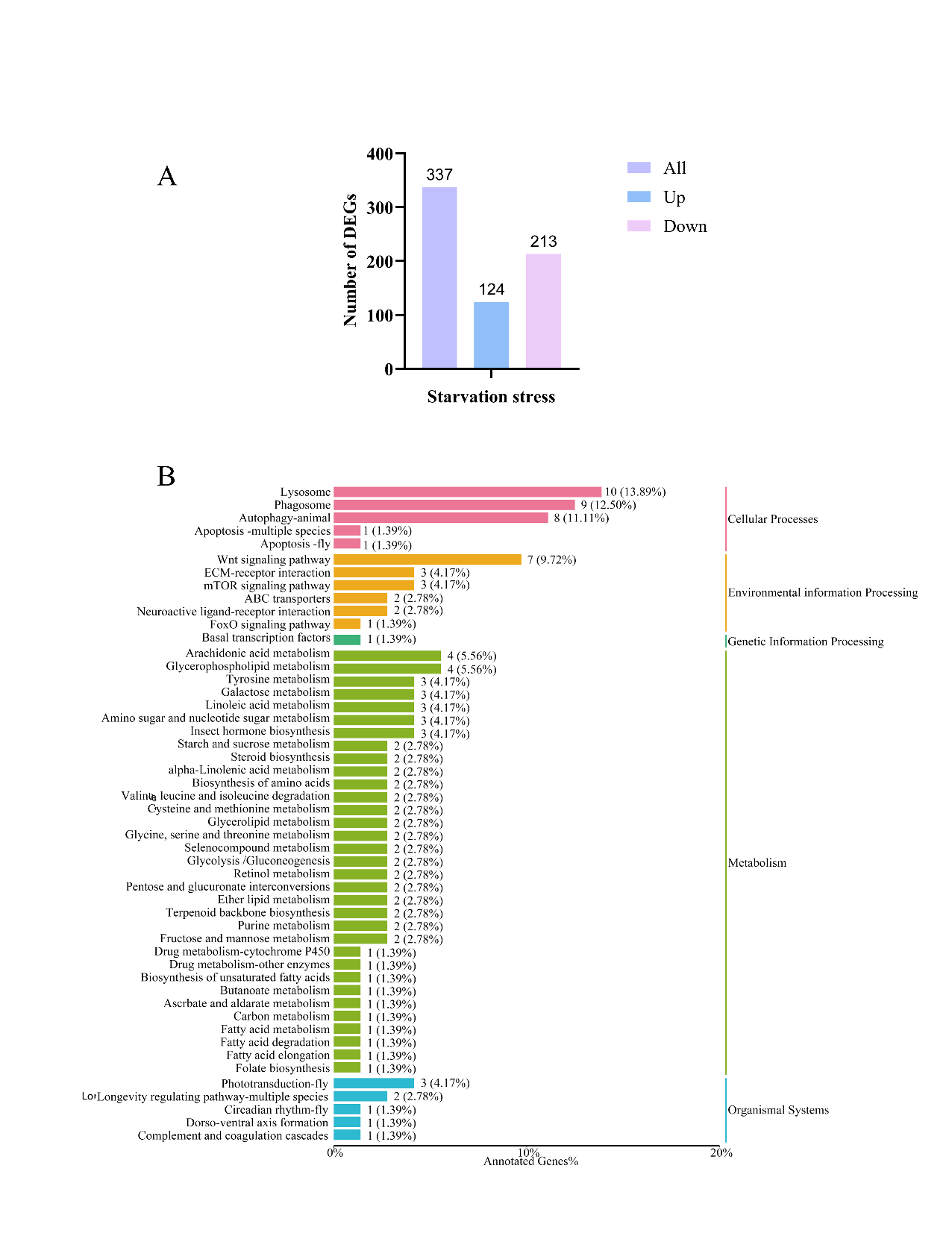
**Fig. S1.** Analysis of DEGs in *H. variegata* under starvation stress. (A) The number of DEGs between the starvation stress and the control groups. (B) KEGG enrichment analysis of DEGs between the starvation stress and the control groups. The X-axis shows the enrichment factor. The sizes of the circle indicate the total number of enriched genes.



**Fig. S2.** GO functional annotations of DEGs of *H. variegata* in the comparison between starvation stress and the control group. (A-C) classified by cellular components (CCs), biological processes (BPs) and molecular functions (MFs).


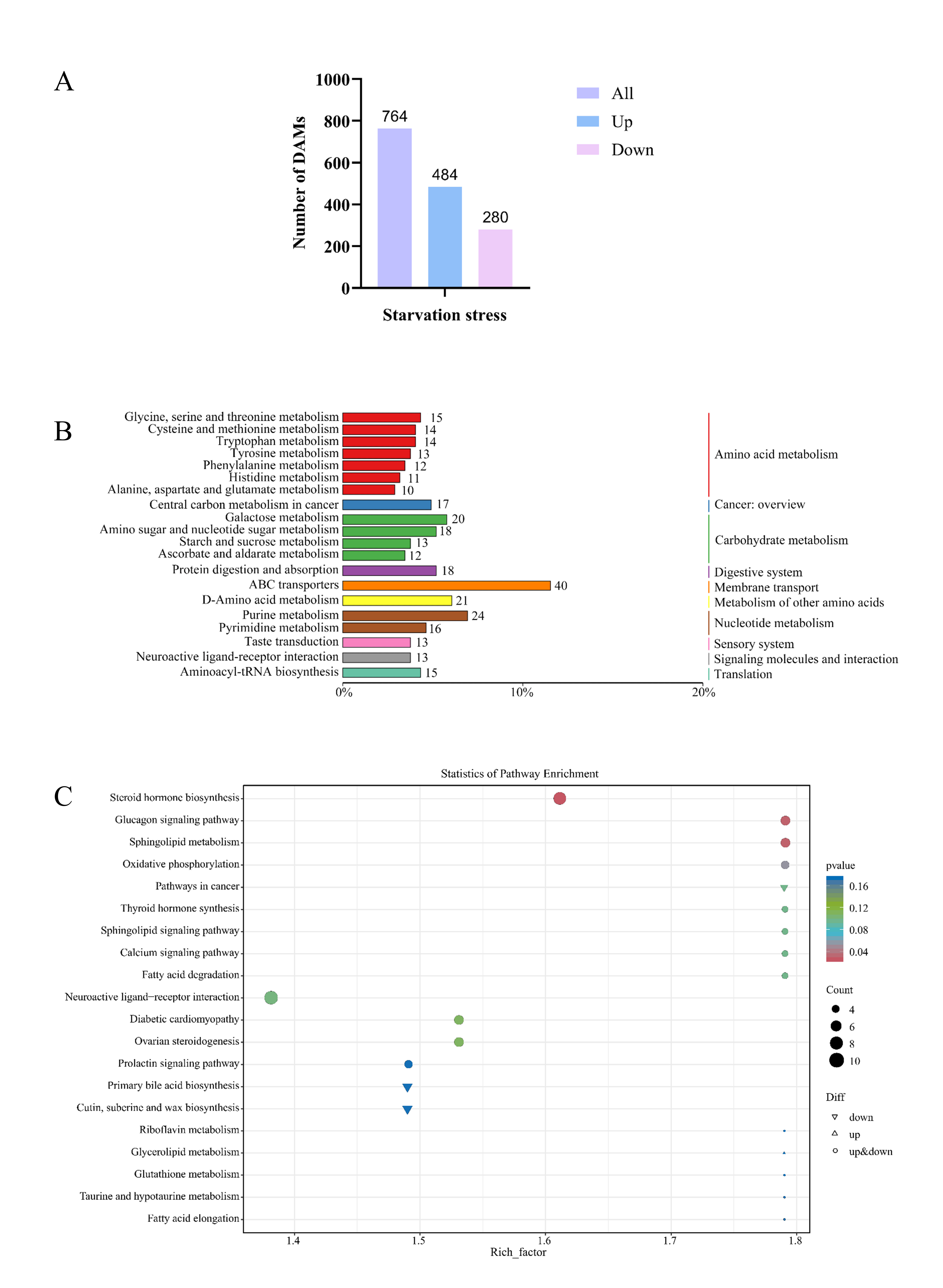
**Fig. S3.** Metabolomic analysis of *H. variegata* under starvation conditions. (A) DAMs of *H. variegata* between the starvation stress and the control groups. (B) The quantity of differential metabolites between the starvation stress and the control group. (C) KEGG enrichment analysis of DAMs between the starvation stress and the control group.

| 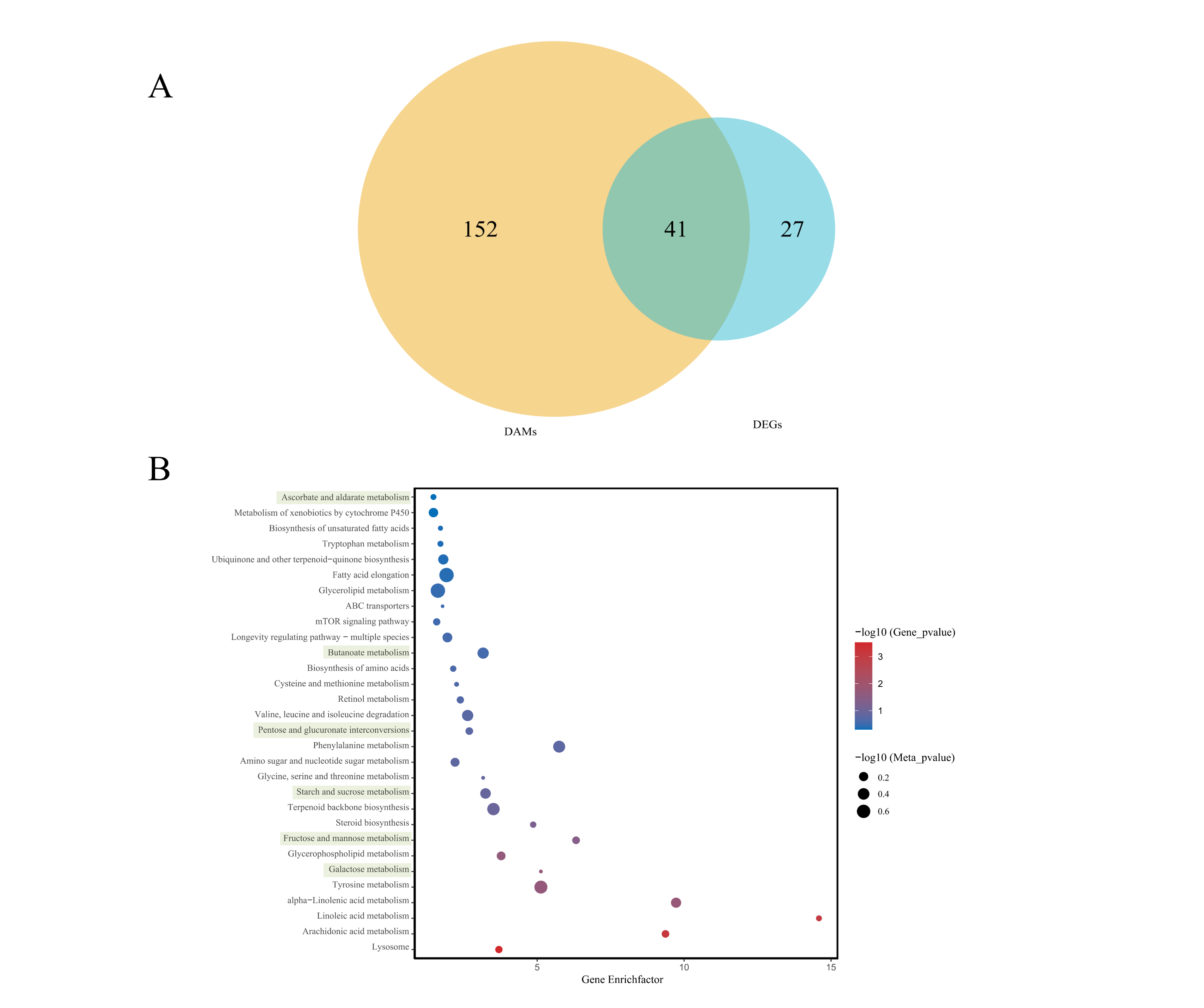 |  |
| --- | --- |

**Fig. S4.** Integrated analysis of DAMs and DEGs of *H. variegata*. (A) Venn diagram showing the number of common and unique pathways in the comparison between starvation stress and the control group. (B) Common pathway showing the degree of enrichment of DAMs in the comparison between starvation stress and the control group.


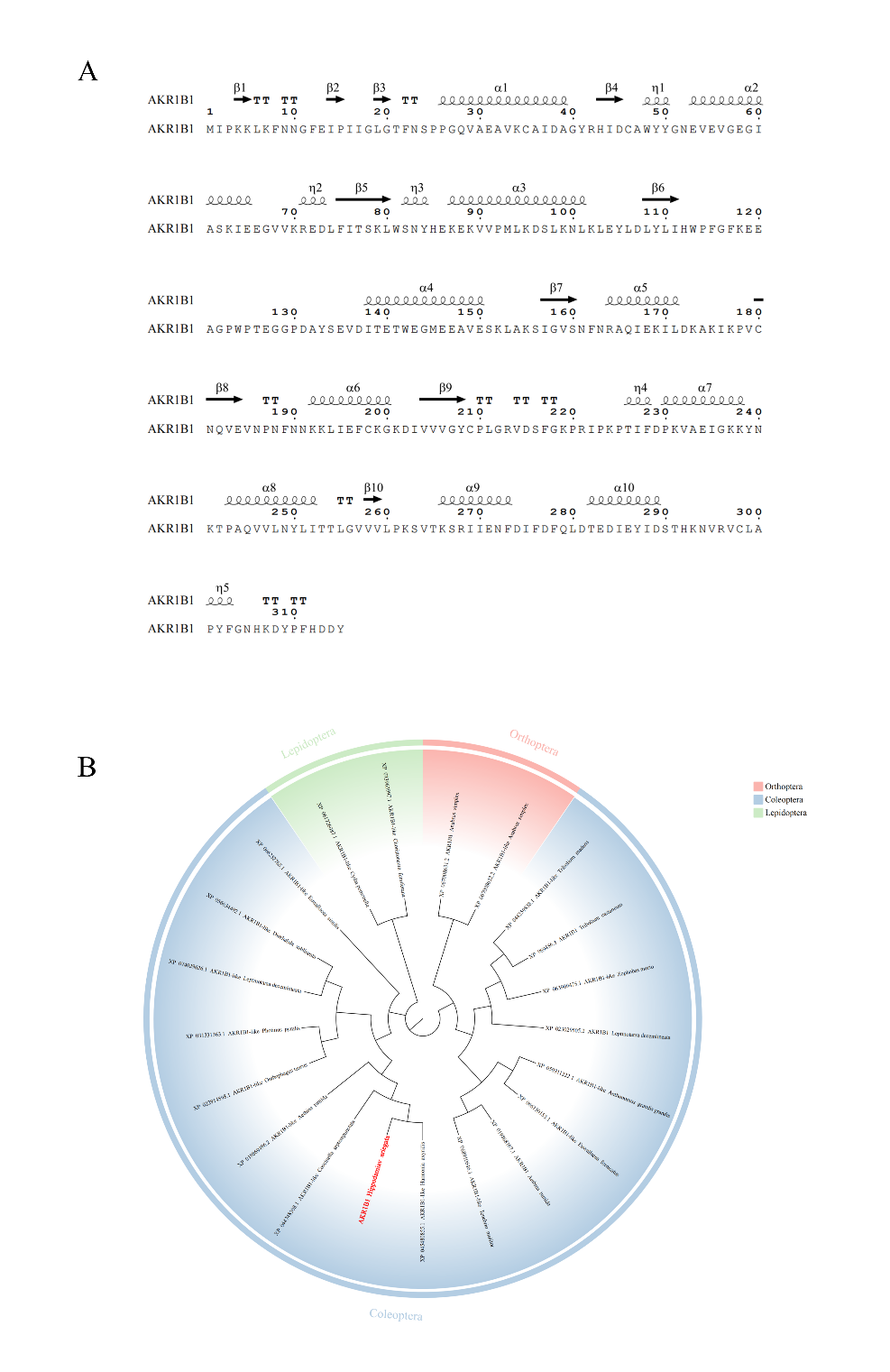
**Fig. S5.** Bioinformatics and phylogenetic analysis of AKR1B1. (A) Secondary structure prediction of AKR1B1 of *H. variegata*. β1~β10: β-folded sheet; α1~α10: α helix; η1~η5: disulfide bond. (B) Phylogenetic analysis of *H. variegata* with other AKR1B1 sequences from other species. The phylogenetic tree is based on amino acid sequences using the neighbor-joining method with a bootstrap of 1000 through MEGA11.0.


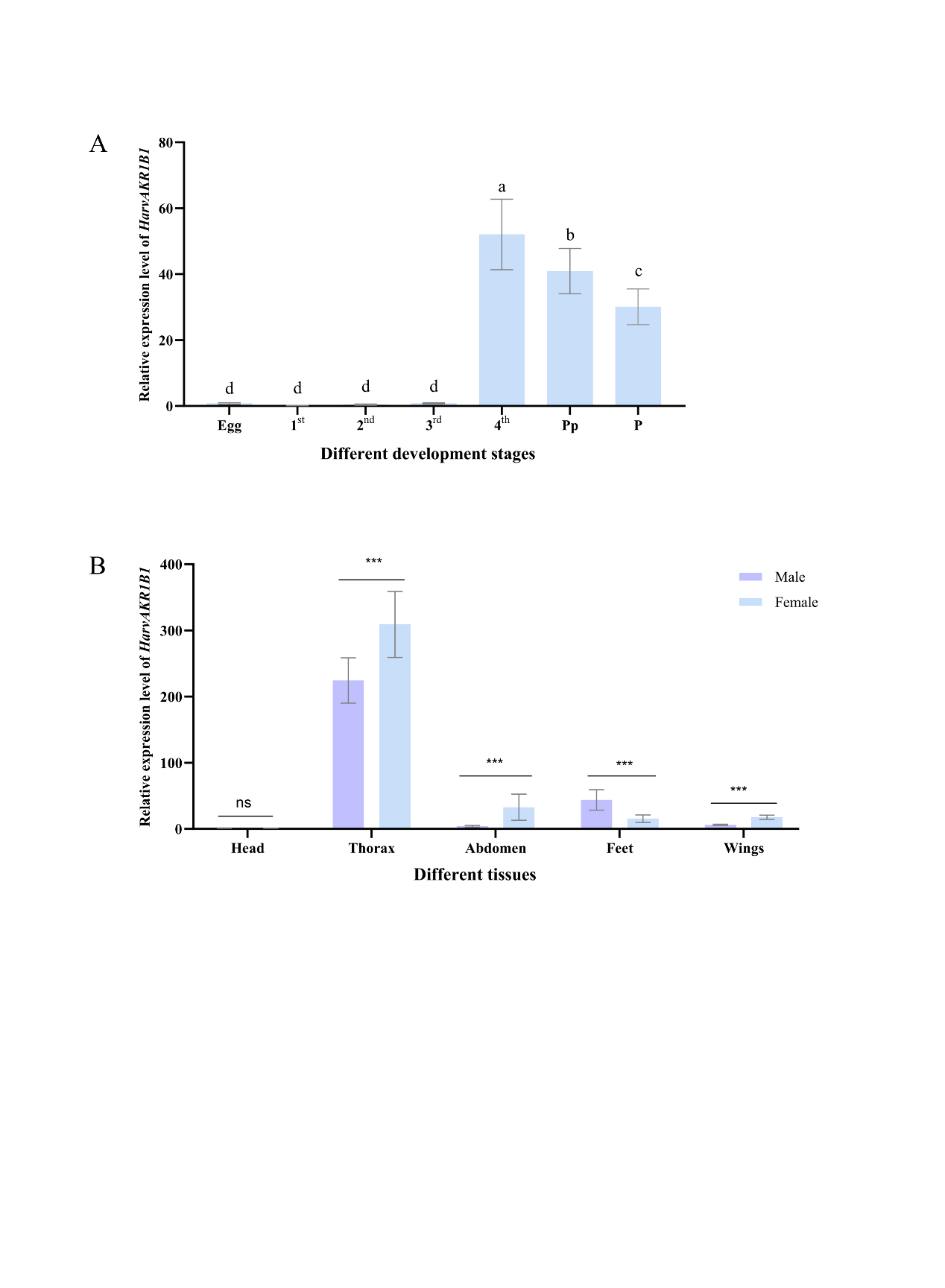
**Fig.** **S6.** Relative expression level of *HvarAKR1B1*. (A) Across different developmental stages of *H. variegata*. (B) In different tissues of *H. variegata*. Asterisks indicate significant differences between females and males (ns, not significant, * *P*<0.05, ** *P*<0.01, *** *P*<0.001, *t*-test). Different lowercase letters indicate significant differences among different developmental stages (one-way ANOVA).
